# Novel systematic processing of cardiac magnetic resonance imaging identifies target regions associated with infarct-related ventricular tachycardia

**DOI:** 10.1093/europace/euae244

**Published:** 2024-09-19

**Authors:** Alba Ramos-Prada, Andrés Redondo-Rodríguez, Ivo Roca-Luque, Andreu Porta-Sánchez, Rachel M A ter Bekke, Jorge G Quintanilla, Javier Sánchez-González, Rafael Peinado, Jose Luis Merino, Matthijs Cluitmans, Robert J Holtackers, Manuel Marina-Breysse, Carlos Galán-Arriola, Daniel Enríquez-Vázquez, Sara Vázquez-Calvo, José Manuel Alfonso-Almazán, Gonzalo Pizarro, Borja Ibáñez, Juan José González-Ferrer, Ricardo Salgado-Aranda, Victoria Cañadas-Godoy, David Calvo, Julián Pérez-Villacastín, Nicasio Pérez-Castellano, David Filgueiras-Rama

**Affiliations:** Novel Arrhythmogenic Mechanisms Program, Centro Nacional de Investigaciones Cardiovasculares (CNIC), Melchor Fernández Almagro 3, 28029, Madrid, Spain; Fundación Interhospitalaria para la Investigación Cardiovascular (FIC), Madrid, Spain; Novel Arrhythmogenic Mechanisms Program, Centro Nacional de Investigaciones Cardiovasculares (CNIC), Melchor Fernández Almagro 3, 28029, Madrid, Spain; Centro de Investigación Biomédica en Red de Enfermedades Cardiovasculares (CIBERCV), Avenida Monforte de Lemos, 3-5, Pabellón 11, Planta 0, 28029, Madrid, Spain; Department of Cardiology, Hospital Clínic, Barcelona, Spain; Centro de Investigación Biomédica en Red de Enfermedades Cardiovasculares (CIBERCV), Avenida Monforte de Lemos, 3-5, Pabellón 11, Planta 0, 28029, Madrid, Spain; Department of Cardiology, Hospital Clínic, Barcelona, Spain; Department of Cardiology, Cardiovascular Research Institute Maastricht (CARIM), Maastricht University Medical Center, Maastricht, The Netherlands; Novel Arrhythmogenic Mechanisms Program, Centro Nacional de Investigaciones Cardiovasculares (CNIC), Melchor Fernández Almagro 3, 28029, Madrid, Spain; Centro de Investigación Biomédica en Red de Enfermedades Cardiovasculares (CIBERCV), Avenida Monforte de Lemos, 3-5, Pabellón 11, Planta 0, 28029, Madrid, Spain; Instituto de Investigación Sanitaria del Hospital Clínico San Carlos (IdISSC), Cardiovascular Institute, Madrid, Spain; Philips Healthcare Iberia, Madrid, Spain; Cardiology Department, Hospital Universitario La Paz, Madrid, Spain; Cardiology Department, Hospital Universitario La Paz, Madrid, Spain; Cardiovascular Research Institute Maastricht (CARIM), Maastricht University, Maastricht, The Netherlands; Philips Research, Eindhoven, The Netherlands; Cardiovascular Research Institute Maastricht (CARIM), Maastricht University, Maastricht, The Netherlands; Department of Radiology and Nuclear Medicine, Maastricht University Medical Center, Maastricht, The Netherlands; Novel Arrhythmogenic Mechanisms Program, Centro Nacional de Investigaciones Cardiovasculares (CNIC), Melchor Fernández Almagro 3, 28029, Madrid, Spain; Centro de Investigación Biomédica en Red de Enfermedades Cardiovasculares (CIBERCV), Avenida Monforte de Lemos, 3-5, Pabellón 11, Planta 0, 28029, Madrid, Spain; Novel Arrhythmogenic Mechanisms Program, Centro Nacional de Investigaciones Cardiovasculares (CNIC), Melchor Fernández Almagro 3, 28029, Madrid, Spain; Centro de Investigación Biomédica en Red de Enfermedades Cardiovasculares (CIBERCV), Avenida Monforte de Lemos, 3-5, Pabellón 11, Planta 0, 28029, Madrid, Spain; Centro de Investigación Biomédica en Red de Enfermedades Cardiovasculares (CIBERCV), Avenida Monforte de Lemos, 3-5, Pabellón 11, Planta 0, 28029, Madrid, Spain; Unidad de Insuficiencia Cardíaca Avanzada y Trasplante Cardiaco, Department of cardiology, Complexo Hospitalario Universitario A Coruña, Instituto de Investigación Biomédica de A Coruña (INIBIC), A Coruña, Spain; Department of Cardiology, Hospital Clínic, Barcelona, Spain; Novel Arrhythmogenic Mechanisms Program, Centro Nacional de Investigaciones Cardiovasculares (CNIC), Melchor Fernández Almagro 3, 28029, Madrid, Spain; Novel Arrhythmogenic Mechanisms Program, Centro Nacional de Investigaciones Cardiovasculares (CNIC), Melchor Fernández Almagro 3, 28029, Madrid, Spain; Cardiology Department, Hospital Ruber Juan Bravo Quiron Salud, UEM, Madrid, Spain; Novel Arrhythmogenic Mechanisms Program, Centro Nacional de Investigaciones Cardiovasculares (CNIC), Melchor Fernández Almagro 3, 28029, Madrid, Spain; Centro de Investigación Biomédica en Red de Enfermedades Cardiovasculares (CIBERCV), Avenida Monforte de Lemos, 3-5, Pabellón 11, Planta 0, 28029, Madrid, Spain; Cardiology Department, IIS-University Hospital Fundación Jiménez Díaz, Madrid, Spain; Centro de Investigación Biomédica en Red de Enfermedades Cardiovasculares (CIBERCV), Avenida Monforte de Lemos, 3-5, Pabellón 11, Planta 0, 28029, Madrid, Spain; Instituto de Investigación Sanitaria del Hospital Clínico San Carlos (IdISSC), Cardiovascular Institute, Madrid, Spain; Instituto de Investigación Sanitaria del Hospital Clínico San Carlos (IdISSC), Cardiovascular Institute, Madrid, Spain; Centro de Investigación Biomédica en Red de Enfermedades Cardiovasculares (CIBERCV), Avenida Monforte de Lemos, 3-5, Pabellón 11, Planta 0, 28029, Madrid, Spain; Instituto de Investigación Sanitaria del Hospital Clínico San Carlos (IdISSC), Cardiovascular Institute, Madrid, Spain; Instituto de Investigación Sanitaria del Hospital Clínico San Carlos (IdISSC), Cardiovascular Institute, Madrid, Spain; Fundación Interhospitalaria para la Investigación Cardiovascular (FIC), Madrid, Spain; Centro de Investigación Biomédica en Red de Enfermedades Cardiovasculares (CIBERCV), Avenida Monforte de Lemos, 3-5, Pabellón 11, Planta 0, 28029, Madrid, Spain; Instituto de Investigación Sanitaria del Hospital Clínico San Carlos (IdISSC), Cardiovascular Institute, Madrid, Spain; Fundación Interhospitalaria para la Investigación Cardiovascular (FIC), Madrid, Spain; Centro de Investigación Biomédica en Red de Enfermedades Cardiovasculares (CIBERCV), Avenida Monforte de Lemos, 3-5, Pabellón 11, Planta 0, 28029, Madrid, Spain; Instituto de Investigación Sanitaria del Hospital Clínico San Carlos (IdISSC), Cardiovascular Institute, Madrid, Spain; Novel Arrhythmogenic Mechanisms Program, Centro Nacional de Investigaciones Cardiovasculares (CNIC), Melchor Fernández Almagro 3, 28029, Madrid, Spain; Centro de Investigación Biomédica en Red de Enfermedades Cardiovasculares (CIBERCV), Avenida Monforte de Lemos, 3-5, Pabellón 11, Planta 0, 28029, Madrid, Spain; Instituto de Investigación Sanitaria del Hospital Clínico San Carlos (IdISSC), Cardiovascular Institute, Madrid, Spain

**Keywords:** Ventricular tachycardia, Radiofrequency ablation, Magnetic resonance imaging, Imaging processing

## Abstract

**Aims:**

There is lack of agreement on late gadolinium enhancement cardiac magnetic resonance (LGE-CMR) imaging processing for guiding ventricular tachycardia (VT) ablation. We aim at developing and validating a systematic processing approach on LGE-CMR images to identify VT corridors that contain critical VT isthmus sites.

**Methods and results:**

This is a translational study including 18 pigs with established myocardial infarction and inducible VT undergoing *in vivo* characterization of the anatomical and functional myocardial substrate associated with VT maintenance. Clinical validation was conducted in a multicentre series of 33 patients with ischaemic cardiomyopathy undergoing VT ablation. Three-dimensional LGE-CMR images were processed using systematic scanning of 15 signal intensity (SI) cut-off ranges to obtain surface visualization of all potential VT corridors. Analysis and comparisons of imaging and electrophysiological data were performed in individuals with full electrophysiological characterization of the isthmus sites of at least one VT morphology. In both the experimental pig model and patients undergoing VT ablation, all the electrophysiologically defined isthmus sites (*n* = 11 and *n* = 19, respectively) showed overlapping regions with CMR-based potential VT corridors. Such imaging-based VT corridors were less specific than electrophysiologically guided ablation lesions at critical isthmus sites. However, an optimized strategy using the 7 most relevant SI cut-off ranges among patients showed an increase in specificity compared to using 15 SI cut-off ranges (70 vs. 62%, respectively), without diminishing the capability to detect VT isthmus sites (sensitivity 100%).

**Conclusion:**

Systematic imaging processing of LGE-CMR sequences using several SI cut-off ranges may improve and standardize procedure planning to identify VT isthmus sites.

## Graphical Abstract

**Graphical Abstract:**
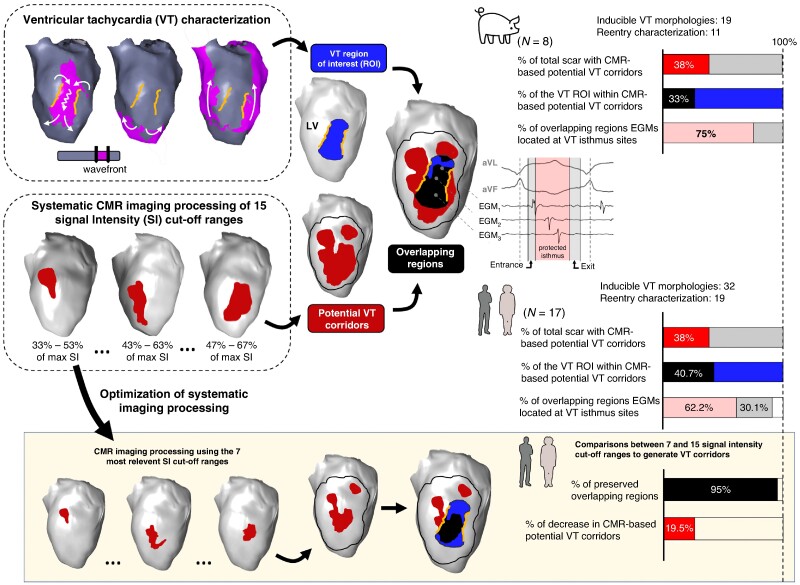

## Introduction

Ventricular tachycardia (VT) characterization using electroanatomic mapping systems during tachycardia enables interventional electrophysiologists to obtain high-density activation maps with multipolar catheters and define the target region for VT termination.^1^ In well-tolerated VT episodes, classical electrophysiological manoeuvres using resetting and entrainment also complement the information obtained on activation maps to further confirm the location of VT isthmuses for radiofrequency delivery and VT termination.^2^ However, complete characterization of VT channels within the scar tissue is difficult and often unmappable due to multiple inducible VT morphologies or haemodynamic instability during VT which requires immediate cardioversion. These common limitations have motivated the description of several mapping strategies during sinus rhythm or ventricular pacing aiming to identify scar regions with potential arrhythmogenicity.^3–7^ Some of these strategies also enable a precise characterization of scar regions prone to clinically relevant reentrant arrhythmias.^3,4^ Notwithstanding, all these strategies require an invasive procedure and time-consuming catheter-based mapping.

Imaging-based strategies have also been proposed as complementary tools to identify the myocardial substrate that may be associated with reentrant VTs.^8–10^ Potential tissue channels on signal intensity (SI) maps from late gadolinium enhancement cardiac magnetic resonance (LGE-CMR) images have shown good correlation with electrograms with delayed components,^8^ which may be associated with electrophysiologically (EP) relevant conducting channels.^11^ More recently, a more detailed analysis on LGE-CMR-derived tissue channels has also shown that specific channel features as protectedness and length may also be associated with arrhythmogenicity.^9,12^ Overall, these and other series have provided evidence that supports the use of pre-procedural imaging to guide VT ablation.^13,14^

Despite rapid development for incorporating cardiac imaging into complex ablation procedures, controversy still remains about relevant processing steps that may substantially affect procedure planning and imaging-derived target regions. The most recent expert consensus report on catheter ablation of ventricular arrhythmia highlights the lack of agreement on the preferable SI cut-off values for assessment of tissue heterogeneity and scar areas.^13^ This makes the selection of cut-off values highly operator-dependent within a wide range of SI options.^8^ Moreover, variability among series supports the premise that a single and universal SI cut-off range may not fit for all cases. Among other factors, imaging acquisition time, heart rate, and imaging quality vary substantially among patients,^15,16^ which may affect the most appropriate range of SI cut-off values to identify potential tissue channels.

Here, we aimed at developing and validating a novel systematic processing approach on LGE-CMR images to identify VT corridors that contain critical VT isthmus sites. We hypothesized that myocardial surface visualization of imaging-based VT corridors using a sequential sweeping of different SI cut-off ranges effectively identifies critical isthmus sites that cannot be detected using a single universal SI cut-off range.

## Methods

### Experimental study

The study included 18 pigs (strain large white) with myocardial infarction in the anterior wall (see Supplementary material online, *Figure S1*) and *in vivo* characterization of the ventricular substrate. Ten to 12 weeks after myocardial infarction, all pigs underwent high-resolution 3D LGE-CMR imaging and an invasive electrophysiological study to identify isthmus sites of reentrant inducible VTs. Only cases with full electrophysiological characterization of at least one of the reentrant VT morphologies were included in the analysis. The animal cohort aimed to provide a highly controlled experimental scenario where LGE-CMR imaging would not be affected by acquisition efficiency on 3D sequences. All procedures in pigs were performed under general anaesthesia (intravenous ketamine 2 mg/kg/h, xylazine 0.2 mg/kg/h, and midazolam i.v. 0.2 mg/kg/h) and were approved by the Comunidad de Madrid (Ref#PROEX097/17). Animal experiments complied with Animal Research: Reporting of In Vivo Experiments (ARRIVE) guidelines.

### Clinical study

Multicentre study that included consecutive patients admitted to the cardiology department with infarct-related VT episodes who were subsidiary for VT ablation. Prospective enrolment started in November 2013 at the Hospital La Paz (Madrid, Spain) and in November 2014 at the Hospital Clínico San Carlos. Two additional centres, the Hospital Clínic (Barcelona, Spain) and the Maastricht University Medical Center (Maastricht, The Netherlands), started the recruitment in October 2018 and November 2021, respectively. Thirty-three patients undergoing 3D LGE-CMR imaging before the ablation procedure were invited to participate. Three candidates were excluded before invasive mapping due to low imaging quality on 3D LGE-CMR sequences. Baseline clinical data were obtained at the time of the CMR study. After the ablation procedure, only patients with electrophysiological identification of VT isthmus sites were included in the analysis. In cases with more than one VT morphology during the ablation procedure, at least one of the VT isthmuses of such morphologies had to be fully characterized based on electrophysiological criteria.^1,2,4^ The ethical committees of the four participant hospitals approved the protocol [Hospital La Paz (Ref#PI-1464), Hospital Clínico San Carlos (Ref#14/246-E_BC), Hospital Clínic (Ref#HCB/2020/0544), and Hospital Maastricht (Ref#2022-3170)]. The study was performed in accordance with the standards of the 2013 revision of the Declaration of Helsinki. All patients signed the informed consent.

### Magnetic resonance imaging acquisition

In pigs, *in vivo* substrate characterization was performed with a Philips Achieva 3T-Tx whole-body scanner equipped with a 32-element and phased-array cardiac coil (Philips Healthcare, Best, The Netherlands), as reported elsewhere.^15,17^ Seven minutes after intravenous contrast injection of 0.2 mmol/kg gadoteric acid (Dotarem, Guerbet, France), 3D LGE-CMR sequences were acquired using an inversion-recovery spoiled turbo field echo (IR-T1TFE) with isotropic resolution of 1.5 × 1.5 × 1.5 mm.

In patients, 3D LGE-CMR sequences were obtained between 7 and 15 minutes after intravenous contrast administration of 0.2 mmol/kg gadoteric acid (Dotarem), gadodiamide-DTPA (Omniscan, Amersham Health), or gadobutrol (Gadovist, Bayer Pharmaceuticals, Germany) depending on the protocol at each participant centre. Acquisition resolutions were 1.5 × 1.5 × 1.5 mm in two centres and 1.6 × 1.6 × 1.6 mm and 1.2 × 1.2 × 1.2 mm in the other two participant centres.

Specific details of CMR sequences for patients and animals are reported in the Supplementary material.

### Ventricular tachycardia mapping in pigs

Invasive procedures were performed using percutaneous endocardial and epicardial access to obtain full anatomical reconstruction of the heart surfaces. A screw-in catheter was positioned in the right ventricle for programmed ventricular stimulation. Endocardial and epicardial geometries were generated using the Carto3 system (Biosense Webster, Diamond Bar, CA) and a 3.5 mm irrigated-tip mapping/ablation catheter (Navistar Thermocool, Biosense Webster). Ventricular tachycardia induction was performed using up to five basic drive cycle lengths (BDCL, S1; 10 pacing beats) at 350, 300, 280, 260, and 250 ms, with a maximum of four coupled extrastimuli (S2, S3, S4, and S5). At each BDCL, the extrastimuli were progressively decremented in 10 ms steps until VT was induced, refractoriness, or a 180 ms coupling interval, as reported elsewhere.^18^ All pacing manoeuvres were synchronized with the intrinsic QRS complex. Haemodynamically stable VT episodes were mapped using activation mapping and entrainment manoeuvres (when possible) with a 3.5 mm tip catheter or a 20-pole steerable mapping catheter (Pentaray, Biosense Webster).^2^ Direct current cardioversion was delivered to restore sinus rhythm in case of ventricular fibrillation or haemodynamic collapse. Ventricular tachycardia maps, intracardiac electrograms, and surface electrocardiogram (ECG) tracings were stored for further offline processing. Further details are provided in the Supplementary material.

### Mapping and ablation procedure in patients

Invasive electrophysiological procedures were performed using percutaneous venous and arterial femoral access to reach the right and left ventricles, respectively. Ventricular geometries were generated using the Carto3 (Biosense Webster, Diamond Bar, CA) or the EnSite NavX mapping system (Velocity, Precision or X, Abbott, Abbott Park, Illinois). Ventricular tachycardia induction was attempted using programmed ventricular stimulation (synchronized with the intrinsic QRS complex) from the right ventricular apex. Haemodynamically tolerated VT episodes were characterized using activation maps and entrainment manoeuvres (when possible). Mapping data were obtained with the ablation catheter (Navistar Thermocool, Biosense Webster; Tacticath or FlexAbility, Abbott) or a 20-pole steerable mapping catheter (Pentaray, Biosense Webster or HD Grid, Abbott).^1,2,19^ In case of haemodynamic instability during VT, the arrhythmia was terminated, and deductive reconstruction of the reentrant circuit associated with the most clinically relevant VT (i.e. documented VT morphology on admission) was performed using pace mapping, as described by de Chillou *et al*.^4^ Radiofrequency energy was delivered at the isthmus sites using the following ablation settings: 30–45 W, temperature limit 40–45°C, and 17–30 mL flow rate. In cases with VT isthmus reconstruction using pace mapping, radiofrequency energy was initially directed at the region between the entrance and exit sites.^4^ Ventricular tachycardia reinduction was performed after RF delivery at the target sites to assess inducibility of clinically relevant VTs and any other morphology. All additional inducible and haemodynamically tolerated VT morphologies were also mapped using activation maps and entrainment manoeuvres. Other haemodynamically instable VT morphologies, only documented during the electrophysiological study, were targeted using substrate ablation based on late potentials and deceleration zones, following the criterion of the physician in charge.^6,8,11^ At the end of the procedure, VT maps, intracardiac electrograms, and surface ECG tracings were stored for further offline processing. More details are provided in the Supplementary material.

### Identification of cardiac magnetic resonance–based potential ventricular tachycardia corridors

**Figure 1:**
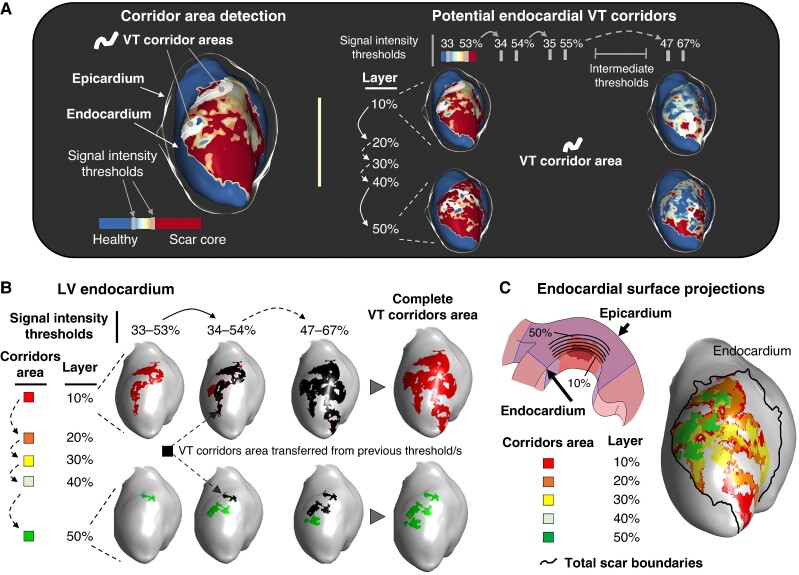
Imaging processing for identification of imaging-based potential subendocardial ventricular tachycardia (VT) corridors. (*A*) Representative workflow for imaging processing using the ADAS 3D software after semiautomatic segmentation of the left ventricle (LV). All VT corridors [using 15 sequential SI cut-off ranges] from 10 to 50% of the myocardial wall thickness were exported for further imaging processing using MATLAB. (*B* and *C*) Computed subendocardial VT corridors from all SI cut-off ranges were projected on the endocardial surface. Overlapping corridor areas from different subendocardial layers (i.e. 10 to 50% of myocardial wall thickness) or SI cut-off ranges were represented on the same area (C). SI, signal intensity.

Clinical and experimental LGE-CMR images were processed using the ADAS3D software (v.2.11.0, Adas3D Medical S.L., Barcelona).^8,9^ Segmented images were processed using a systematic approach to obtain imaging-derived potential VT corridors from 15 different and sequential SI cut-off ranges. Such 15 SI cut-off ranges were selected from the most commonly reported in the literature,^8,15,20–22^ starting at 33% (for heterogeneous scar) and 53% (for dense scar) of the maximum SI detected with the ADAS3D software. The starting point at 33% was selected after an initial analysis, in which SI cut-off ranges <33–53% showed minimal contribution (6.06%) to imaging-derived potential VT corridors (see Supplementary material online, *Figure S2*). For each SI cut-off range, the software computed all the potential VT corridors from the endocardium to the epicardium across nine myocardial layers (i.e. from 10 to 90% of the myocardial wall thickness) (*Figure 1A*). The process was repeated for each SI cut-off range until completion of 15 sequential cut-off ranges (*Figure 1A*). Then, VT corridors data from each cut-off range of SI values were exported and further processed in MATLAB. More in detail, all the computed potential subendocardial VT corridors from the first five myocardial layers (10 to 50% of the myocardial wall thickness) were projected on the endocardial surface to generate a footprint of potential VT corridors within the subendocardial half of the myocardial wall (*Figure* *1B* and *C*). The rest of potential VT corridors from the remaining four myocardial layers (60 to 90% of the myocardial wall thickness) were projected on the epicardial surface (or right side of the interventricular septum, if applicable) (see Supplementary material online, *Figure S3A–C*). All custom-written code is available upon request.

### Delineation of electrophysiologically defined ventricular tachycardia regions of interest and registration onto cardiac magnetic resonance geometries for comparisons

**Figure 2:**
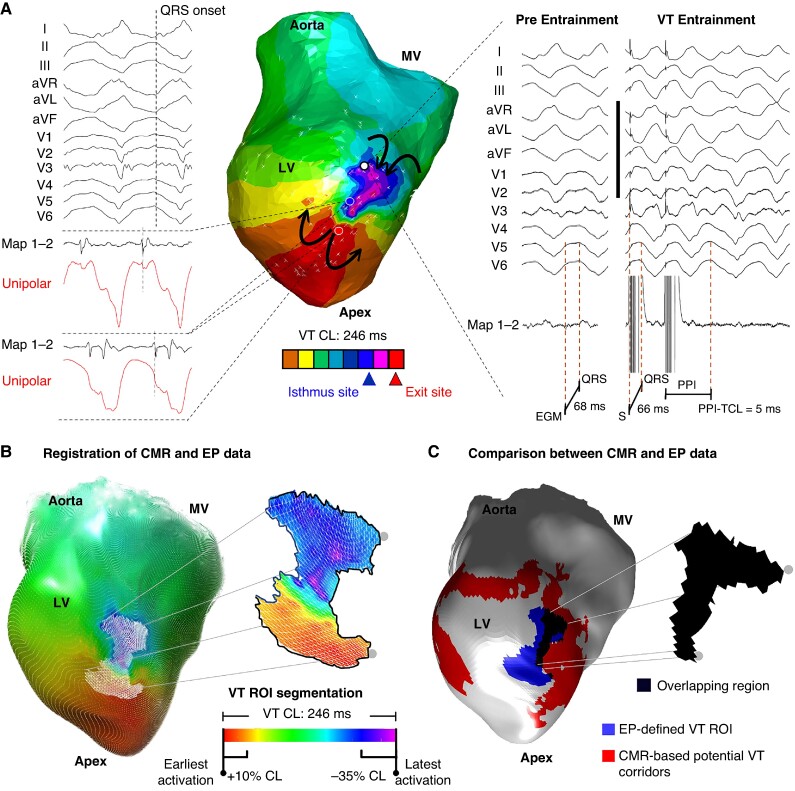
Electrophysiological identification and segmentation of ventricular tachycardia regions of interest. (*A*) Sample electrophysiological characterization of a reentrant ventricular tachycardia (VT) circuit in the pig model. The activation map and entrainment manoeuvres were used to identify the VT isthmus. (*B* and *C*) Segmentation of the electrophysiologically (EP)-defined VT region of interest (ROI) on a sample activation map. The VT ROI is registered onto the left ventricular (LV) cardiac magnetic resonance (CMR) geometry for further comparisons with imaging-based potential VT corridors (*C*). CL, cycle length; EGM, electrogram; MV, mitral valve; PPI, post-pacing interval.

Activation maps were used as the reference mapping data to delineate the VT region of interest (ROI).^2^ The reference for assigning activation times during VT was the QRS onset (*Figure 2A*). The VT ROI was defined as the area with activation times from the latest activation point to −35% of the tachycardia CL and from the earliest activation point to +10% of the tachycardia CL (*Figure 2B*), including also the junction between the earliest and latest activation points. In VTs only characterized with pace mapping (see Supplementary material online, *Figure S4A*), exit and entrance sites areas were connected using the coordinates containing the estimated central isthmus. In VTs only characterized with entrainment, the locations defining the VT isthmus were used as individual points connected among them.

The EP-defined VT ROIs from the mapping data were registered onto the left ventricular CMR geometry for further comparisons with CMR-based potential VT corridors (*Figure* *2B* and *C*). The registration process started with the identification of specific anatomical landmarks from different spatial planes in both the electroanatomical mapping and CMR geometries. For the left ventricle, we used the aortic root, the left ventricular apex, and the septal and lateral part of the mitral annulus. The registration was done using the mesh registration module of the Amira-Avizo software 2021 (Thermo Fisher Scientific, Berlin) (see also the Supplementary material). Electrophysiologically defined VT ROIs were visualized on the endocardial surface, or epicardial, if the VT ROI involved the epicardial layers of the left ventricle. Overlapping regions between EP-defined VT ROIs and CMR-based potential VT corridors were quantified and colour-coded in black (*Figure 2C*).

### Clinical follow-up

All patients underwent clinical follow-up annually and following the physician’s in charge criterion, in case that shorter follow-up intervals were needed. Ventricular tachycardia recurrences were defined as follows: (i) any sustained VT episode (duration ≥ 30 s or with haemodynamic collapse if lasted <30 s) detected on new hospital admissions or (ii) any VT episode detected on the implantable cardioverter defibrillator requiring device therapy (anti-tachycardia pacing or DC shock) or reaching the 30 s duration threshold/hemodynamic collapse.

### Statistical analysis

Data are expressed as median and interquartile range for quantitative variables and number and percentage for qualitative variables. Data normality was assessed with the Shapiro–Wilk test. Statistical significance was assessed by the *t*-test or the Mann–Whitney/Wilcoxon test, as appropriate. A *P* < 0.05 was considered statistically significant for differences in group comparisons. All statistical analyses were performed with GraphPad Prism8 (California, USA) and custom-made software in MATLAB.

## Results

### Imaging-based corridors in pigs include functionally relevant ventricular tachycardia isthmus sites

**Figure 3:**
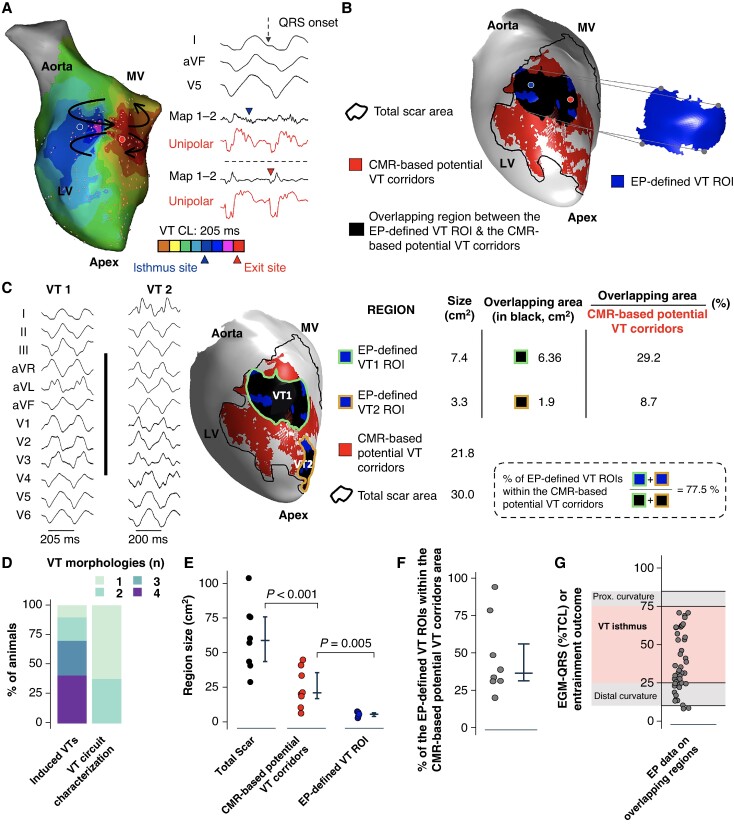
Imaging-based corridors contain isthmus sites for ventricular tachycardia (VT) maintenance in pigs. (*A*) Sample activation map and representative electrograms for reentry characterization of one VT morphology in a pig with infarct-related substrate. (*B*) Simultaneous visualization of cardiac magnetic resonance (CMR)-based potential VT corridors and the electrophysiologically (EP)-defined VT region of interest (ROI) of the VT shown in *A*. (*C*) Left: 12-lead ECG tracings of two fully characterized VT morphologies in the same heart as in *A* and *B*. Centre: visualization of the two EP-defined VT ROIs on the CMR geometry of the left ventricle (LV). Right: quantifications of the EP-defined VT ROIs, CMR-based potential VT corridors, and total scar area for the same heart. (*D*) Percentage of pigs with a specific number of inducible VT morphologies during the EP study and those with full characterization of the reentrant VT circuit. (*E*) Quantification of the areas of total scar, CMR-based potential VT corridors, and EP-defined VT ROIs in the eight pigs included in the analysis. The boundaries of the total scar area were defined using the first cut-off range of signal intensity (SI) values (33% for heterogeneous scar and 53% for dense scar). (*F*) Percentage of the area of EP-defined VT ROIs within the CMR-based potential VT corridors area. (*G*) Electrophysiological characterization of electrograms within overlapping regions. CL, cycle length; MV, mitral valve.

The analysis included all animals with full electrophysiological characterization of at least one reentrant VT morphology. A sample case with electrophysiological characterization of the isthmus sites of two VT morphologies is shown in *Figure 3A–C*. In 8 of 18 pigs of the experimental series, a total of 11 VT morphologies out of 19 inducible VTs were fully characterized during the invasive electroanatomical mapping procedures (*Figure 3D*). In such 11 VT morphologies, VT isthmuses were consistently identified in the endocardium. In the remaining 10 pigs, early haemodynamic collapse upon VT induction (*n* = 6) or reproducible ventricular fibrillation induction (*n* = 4) after programmed ventricular stimulation did not enable electrophysiological characterization of VT isthmuses. Baseline animal data and VT characterization data are shown in Supplementary material online, *Table S1*.

In pigs, activation maps included electrograms covering 84.9% (68.5, 93.2%) of the VT CL. Further entrainment manoeuvres for VT characterization (*Figure 2A*) were also possible in three VT morphologies from three animals (see Supplementary material online, *Table S1*). The analysis of all cases showed that the area of CMR-based potential VT corridors was significantly smaller than the total scar area [21.1 cm^2^ (14.9, 37.4 cm^2^) vs. 58.8 cm^2^ (42.6, 76.0 cm^2^) respectively, *P* < 0.001; *Figure 3E*]. Overall, CMR-based potential VT corridors represented 38.0% (29.6, 49.2%) of the total scar area. The 11 EP-defined VT ROIs were also significantly smaller than the area of CMR-based potential VT corridors [5.6 cm^2^ (3.9, 6.4 cm^2^) vs. 21.1 cm^2^ (14.8, 37.4 cm^2^), respectively, *P* = 0.005; *Figure 3E*].

All VT morphologies with full characterization of the reentrant circuit showed overlapping regions with CMR-based potential VT corridors. More specifically, 33.2% (27.9, 61.7%) of the total area of EP-defined VT ROIs was located within regions of CMR-based potential VT corridors (*Figure 3F*). More importantly, 77% of the electrograms within the overlapping regions were located at VT isthmus sites, with the remaining 23% located at the proximal and distal curvatures (*Figure 3G*).

### Imaging-based corridors in patients undergoing ventricular tachycardia ablation include isthmus sites

**Figure 4:**
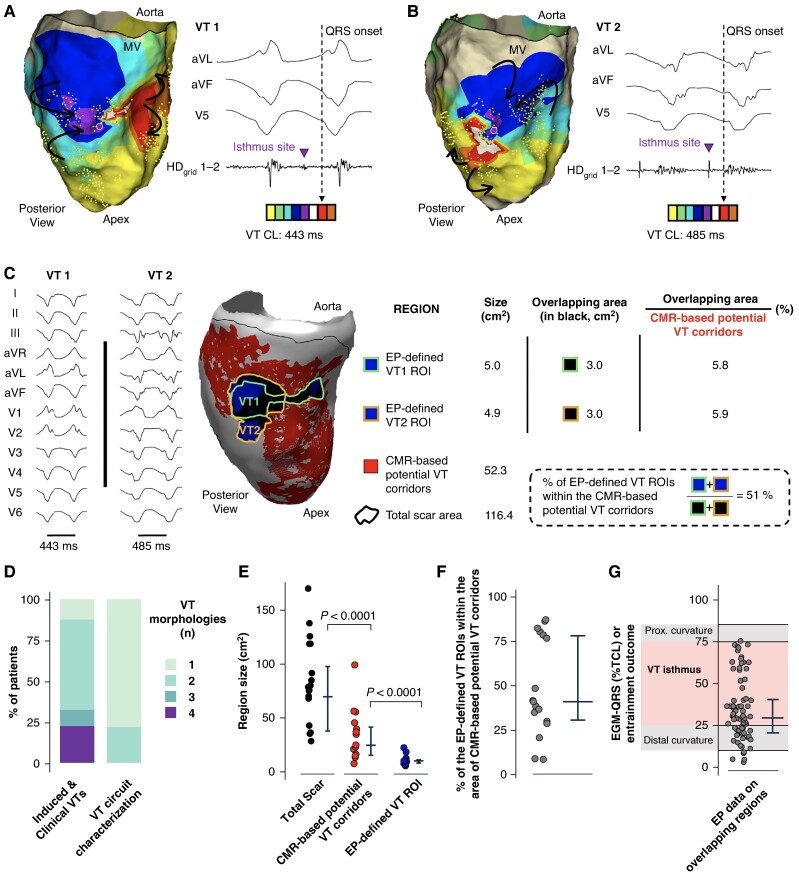
Imaging-based corridors contain isthmus sites for ventricular tachycardia (VT) maintenance in patients. (*A* and *B*) Sample activation maps and representative electrograms for reentry characterization of two VT morphologies in a patient with ischaemic cardiomyopathy. (*C*) Left: 12-lead ECG tracings of two fully characterized VT morphologies in the same heart as in *A* and *B*. Centre: visualization of the two electrophysiologically (EP)-defined VT regions of interest (ROIs) on the cardiac magnetic resonance (CMR) geometry of the left ventricle (LV). The areas of imaging-based potential VT corridors are shown in red. Right: quantifications of the EP-defined VT ROIs, CMR-based potential VT corridors, and total scar area for the same heart. (*D*) Percentage of patients with a specific number of inducible VT morphologies during the electrophysiological study and those with full characterization of the reentrant VT circuit. (*E*) Quantification of the areas of total scar, CMR-based potential VT corridors, and EP-defined VT ROIs in the 17 patients included in the analysis. (*F*) Percentage of the area of EP-defined VT ROIs within the CMR-based potential VT corridors area. (*G*) Electrophysiological characterization of electrograms within overlapping regions. CL, cycle length; MV, mitral valve.

In 17 of 30 study participants, the invasive mapping procedure enabled full electrophysiological characterization of at least one reentrant VT morphology (see Supplementary material online, *Figure S5*). Baseline patient characteristics and medical treatment are shown in *Table 1*. *Figure 4A–C* show representative activation maps for VT characterization of two reentrant VT circuits from the same patient and the comparison of CMR-based potential VT corridors with such EP-defined VT ROIs.

**Table 1:** Baseline patient characteristics and medical treatment

	Patients (*n* = 17)
Baseline characteristics	
Male, *n* (%)	16 (94.12)
Age (years)	69.5 (64.9, 74.5)
Weight (kg)	78 (66.8, 84.3)
Ischaemic cardiomyopathy *n* (%)	17 (100)
Left ventricular ejection fraction (%)	35.0 (31.8, 40)
Hypertension, *n* (%)	12 (70.6)
Diabetes, *n* (%)	6 (35.3)
Smoking	
Current smoker *n* (%)	3 (17.6)
Former smoker *n* (%)	12 (70.6)
Infarcted territory	
Anterior, *n* (%)	6 (35.3)
Inferior, *n* (%)	4 (23.5)
Posterior, *n* (%)	4 (23.5)
Lateral, *n* (%)	9 (53)
Medical treatment	
ACEis/ARBs, *n* (%)	15 (88.2)
β-Blockers, *n* (%)	16 (94.1)
Antiarrhythmic drugs, *n* (%)	7 (41.2)
Antiaggregation, *n* (%)	13 (76.5)
Oral anticoagulation, *n* (%)	6 (35.3)
MRAs, *n* (%)	6 (35.3)
Statins, *n* (%)	15 (88.2)

Values are expressed as median and interquartile ranges and *n* (%), as appropriate.

ACEis, angiotensin converting enzyme inhibitors; ARBs, angiotensin II receptor blockers; MRAs, mineralocorticoid receptor antagonists.

Ventricular tachycardia isthmuses were characterized in 19 VT morphologies out of 32 inducible VTs (*Figure 4D*) using activation maps (*n* = 9), entrainment (*n* = 6), or pace mapping (*n* = 12) (*Table 2*). Activation maps included electrograms covering 83.0% (71.4, 90.1%) of the VT CL. None of the patients required epicardial access. Left and right ventricular mapping was performed in three patients. In fact, in two patients, the VT isthmus involved the interventricular septum on the endocardial side of the right ventricle, which required the complementary data of the left ventricular CMR-based potential VT corridors between 60 and 90% of the myocardial wall thickness.

**Table 2:** Electrophysiological VT characterization and ablation outcomes

Patients (*n* = 17)	
VT characterization	
LGE-CMR imaging before VT mapping (days)	1 (1, 4.2)
Inducible VTs, *n*	32
VTs with characterization of the reentrant circuit, *n* (%)	19 (59.3%)
VT cycle length (ms)	337.8 (294.3, 400.0)
VT morphologies, *n*	2.0 (1.0, 2.0)
Mapping strategy for characterization of the VT circuit	
Entrainment, *n* (%)	6 (31.5%)
Activation map, *n* (%)	9 (47.3%)
Pace mapping, *n* (%)	12 (63.1%)
VT reentrant circuit characterized with >1 mapping strategy	7 (36.8%)
Activation map and pace mapping	3 (15.8%)
Entrainment and pace mapping	1 (5.2%)
Entrainment and activation map	2 (10.5%)
Entrainment, activation map, and pace mapping	1 (5.2%)
Valid mapping points on activation maps, *n*	321 (117, 476)
Ablation outcomes and complications	
Radiofrequency energy delivery (min)	21.6 (13.8, 32.5)
VT termination during radiofrequency delivery, *n* (%)	7 (36.8)
Non-inducibility of any VT morphology after ablation, *n* (%)	14 (82.3)
VT recurrences of any morphology at 1 year, *n* (%)	1 (5.9)
Antiarrhythmic drugs at 1 year after ablation, *n* (%)	7 (41.2)
In-hospital major procedure-related complications:	2 (11.8)
Stroke, transient ischaemic attack, *n* (%)	0 (0)
In-hospital mortality, *n* (%)	0 (0)
Pericardial effusion requiring pericardial drainage, *n* (%)	0 (0)
Groin haematoma requiring blood transfusion	1 (5.9)
Atrio-ventricular node block, *n* (%)	1 (5.9)

Values are expressed as median and interquartile ranges and *n* (%), as appropriate.

LGE-CMR, late gadolinium enhancement cardiac magnetic resonance; VT, ventricular tachycardia.

In patients, the area of CMR-based potential VT corridors was also significantly smaller than the total scar area [20.3 cm^2^ (9.7, 37.2 cm^2^) vs. 72.5 cm^2^ (37.3, 90.3 cm^2^), respectively, *P* < 0.0001; *Figure 4E*]. Cardiac magnetic resonance–based potential VT corridors represented 38.0% (23.7, 48.7%) of the total scar area. The EP-defined VT ROIs were also significantly smaller than the area of CMR-based potential VT corridors [4.67 cm^2^ (2.5, 6.0 cm^2^) vs. 20.3 cm^2^ (9.7, 37.2 cm^2^), respectively, *P* < 0.0001; *Figure 4E*]. Similarly to the experimental data in pigs, 40.7% (28.2, 79.1%) of the area of the EP-defined VT ROIs was within regions of CMR-based potential VT corridors (*Figure* *4F*). Moreover, 61% of electrograms within the overlapping regions were located at VT isthmus sites, with the remaining 39% located at the proximal and distal curvatures (*Figure 4G*).

### Electrophysiologically guided ventricular tachycardia ablation does not require targeting all imaging-based ventricular tachycardia corridors

**Figure 5:**
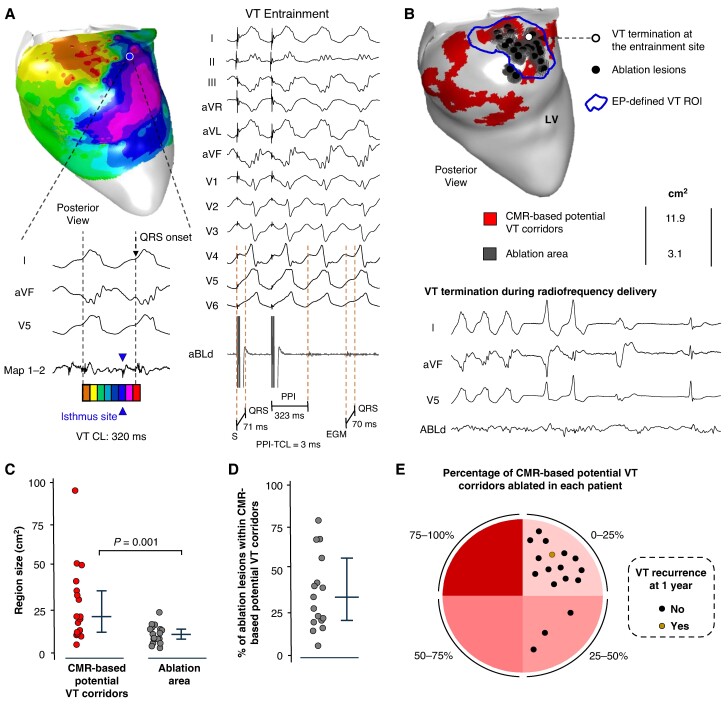
Ablation results and ventricular tachycardia (VT) recurrences during the follow-up. (A) Sample activation map and entrainment manoeuvres to identify the VT isthmus site in a patient with electrophysiologically (EP) characterization of a reentrant VT circuit. (*B*) Upper panel: sample ablation lesions (in black) and estimated ablated area (in dark grey) for each lesion (diameter: 6 mm) in the sample case shown in *A*. Bottom panel: VT termination during radiofrequency delivery at the anatomical site indicated on the upper panel (white circle with black border). (*C*) Comparison of cardiac magnetic resonance (CMR)-based potential VT corridors with the ablated area. (*D*) Percentage of ablation lesions located within CMR-based potential VT corridors. (*E*) Pie chart showing the percentage of CMR-based potential VT corridors ablated for each patient of the series. Dots in the chart indicate individual patients. Black dots indicate patients without VT recurrences of any morphology at 1 year of follow-up. The brown dot in the chart indicates the only patient with documented VT recurrence at 1 year of follow-up (also the sample case shown in *A* and *B*. LV, left ventricle; PPI, post-pacing interval; ROI, region of interest; TCL, tachycardia cycle length.

Radiofrequency delivery at isthmus sites was applied during tachycardia in seven VT morphologies, which demonstrated VT termination in all of them. Sample cases are shown in *Figure* *5A* and *B* and Supplementary material online, *Figure S5*. In the rest of VT morphologies with characterization of the reentrant circuit (*n* = 12), the ablation was performed during sinus rhythm at the target isthmus sites. The ablation was complemented with additional radiofrequency lesions at regions with late potentials and deceleration zones based on the criterion of the operator in charge. After completing the ablation, programmed ventricular stimulation demonstrated non-inducibility of any VT morphology in 14 patients (82.3%). The ablated region was significantly smaller than the area of CMR-based potential VT corridors [9.8 cm^2^ (6.5, 12.9 cm^2^) vs. 20.4 cm^2^ (9.7, 37.2 cm^2^), *P* < 0.0001; *Figure 5C*]. Moreover, 33.4% (19, 59.6%) of ablation lesions were within regions of CMR-based potential VT corridors (*Figure 5D*), which potentially eliminated 12.3% (5.6, 19.8%) of the initial area of imaging-derived corridors (*Figure 5E*).

Ventricular tachycardia recurrences were only documented in one patient after 1 year of follow-up (*Figure 5E*). In this patient, the documented VT morphology during the follow-up was different from the ablated one in the index procedure. The specific case is shown in *Figure* *5A* and *B*. Ablation data, outcomes, and major procedure-related complications are reported in *Table 2*.

### Optimization of imaging processing preserves ventricular tachycardia isthmus site detection and increases specificity

**Figure 6:**
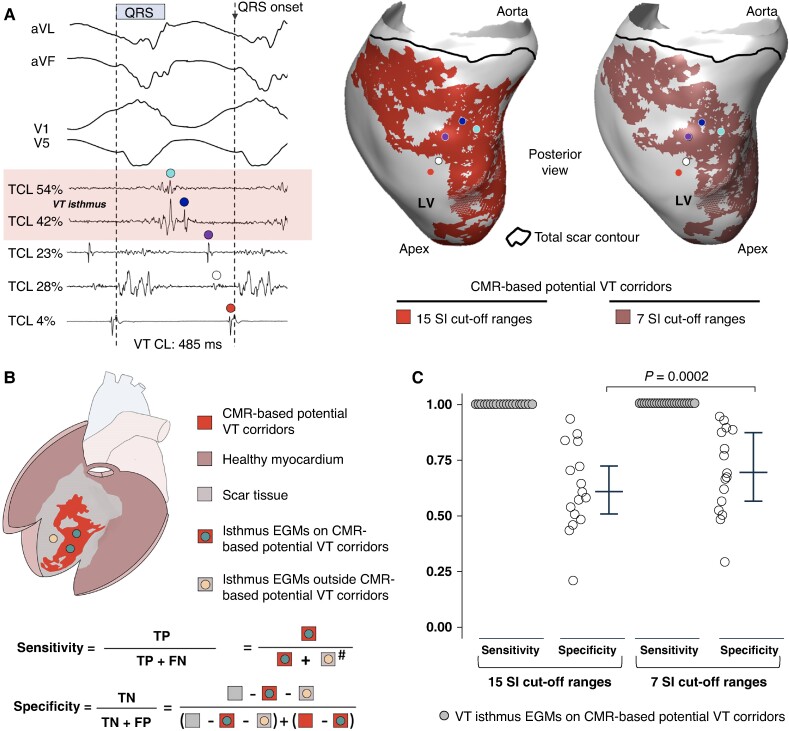
Sensitivity and specificity of imaging-based ventricular tachycardia (VT) corridors to detect isthmus sites. (*A*) Left: sample tracings during VT with colour-coded dots that indicate intracardiac electrograms (EGMs) at different activation times relative (%) to the tachycardia cycle length (CL). The QRS onset was the reference to assign activation times. Colour-coded dots indicate different EGM-QRS times and their relative (%) activation time respect to the tachycardia CL. Middle and right: spatial positioning of colour-coded EGMs on the endocardial surface of the left ventricle (LV) with the corresponding imaging-derived potential VT corridors using 15 (middle) and 7 (right) signal intensity (SI) cut-off ranges. (*B*) Heart scheme showing healthy and infarct-related regions with their respective labels. Sensitivity and specificity values were calculated as indicated. (C) Sensitivity and specificity values of systematic imaging processing using 15 and 7 SI cut-off ranges to detect functionally relevant VT regions of interest (ROIs) containing VT isthmus sites. Cumulative data are shown as median and interquartile range. CMR, cardiac magnetic resonance; FN, false negative; FN, false positive; TN, true negative; TP, true positives. ^#^Ishtmus EGMs outside CMR-based potential VT corridors were considered ‘0’ if interdependent isthmus EGMs (in other position of isthmus sites) were also detected on CMR-based potential VT corridors.

The ablation results and follow-up data support the notion that some CMR-based potential VT corridors may be not functionally relevant. Systematic imaging processing using the seven most relevant SI cut-off ranges, defined as those among patients that provided the largest normalized overlapping area between EP-defined VT ROIs and CMR-based potential VT corridors (see Supplementary material online, *Figure S6*, for details), decreased the area of CMR-based potential VT corridors to a significantly larger extent than the overlapping region between EP-defined VT ROIs and CMR-based potential VT corridors [19.5% (15.6, 39.2%) vs. 5.0% (0.7, 24.2%), respectively, *P* = 0.04; *Figure 6A* and Supplementary material online, *Figure S7A*]. Moreover, this approach preserved a median of 95% of the overlapping area between the VT ROIs of fully characterized VT morphologies and the area of CMR-based potential VT corridors (see Supplementary material online, *Figure S7A* and *B*). The latter was associated with an increase in specificity from a median of 62%, using 15 SI cut-off ranges, to 70% using 7 SI cut-off ranges. This did not affect the capability to identify the EP-defined VT ROI (sensitivity 100%; *Figure* *6B* and *C*). However, further decrease in the number of SI cut-off ranges (to 5, 3, and 1) used for imaging processing nulled the possibility to detect the EP-defined VT ROIs for all patients (see Supplementary material online, *Figures S7A* and *B* and *S8*).

In the experimental animal model, the same approach of decreasing the number of SI cut-off ranges for imaging processing also showed a substantial decrease in the area of CMR-based potential VT corridors (see Supplementary material online, *Figure S9A* and *B*). However, unlike patients, in pigs, the use of seven SI cut-off ranges already nulled the overlapping region between EP-defined VT ROIs and CMR-based potential VT corridors for one case (see Supplementary material online, *Figure S9A* and *B*). Additional decrease in the number of SI cut-off ranges (5, 3, and 1) for systematic imaging processing further affected the capability to detect EP-defined VT ROIs on imaging-derived corridors (see Supplementary material online, *Figures S9A* and *B* and *S10*).

## Discussion

The study shows that systematic processing of several SI cut-off ranges from 3D LGE-CMR images provides a global assessment of the infarct-related substrate and effectively identifies myocardial scar regions associated with VT isthmus sites. Individual-specific variability on the SI cut-off range (or ranges) that identifies the functionally relevant VT substrate does not allow establishing a single and universal SI cut-off range valid for all patients. An ablation approach targeting all imaging-based potential VT corridors derived from 15 SI cut-off ranges will be less specific than targeting and ablating only functionally relevant sites after complete electrophysiological characterization of well-tolerated VT episodes. Notwithstanding, an optimized imaging processing strategy using the seven most relevant SI cut-off ranges among patients increases specificity, without affecting the capability to identify the EP-defined VT ROIs.

A relevant step to identify CMR-based potential VT corridors is the selection of a SI cut-off range before imaging processing. This is challenging, and there is lack of a general rule to aid operators on selecting one cut-off value and not others within the range of the most commonly reported in the literature.^8,15,20–22^ In fact, clinical series of patients undergoing VT ablation have shown variability in the selection of a specific SI cut-off range for substrate characterization.^8,9,15^ Moreover, some series have reported a wide window of SI cut-off ranges potentially selected among patients within the same study,^8,12^ without a clear criterion to select and apply a specific SI threshold in a given patient. Our results highlight such difficulties to select a specific SI cut-off range. Indeed, a single and universal SI cut-off range will not be valid for all patients to identify the functionally relevant VT substrate. This was especially evident in the animal cohort, in which the SI cut-off range that enabled the identification of the functionally relevant VT substrate was not reproducible among animals despite highly reproducible LGE-CMR studies. From the foregoing, it is evident that systematic processing of several SI cut-off ranges may overcome limitations associated with manual selection of a single SI cut-off range. Importantly, our results suggest that the right balance, between decreasing false positive imaging-based potential VT corridors and not missing true positive regions associated with VT isthmus sites, seems to be on using the seven most relevant SI cut-off ranges among cases.

Overall, the study contributes to address major gaps for imaging integration into routine procedure planning in patients undergoing VT ablation.^13^ First, the systematic and sequential scanning of several SI cut-off ranges prevents selection bias problems associated with manual selection of a specific SI range. This also opens the opportunity for a more standardized assessment of tissue heterogeneity and scar areas on LGE-CMR images of patients undergoing VT ablation. Second, the imaging processing approach developed in this study enables to represent and visualize in a simplified manner a vast amount of information from different myocardial layers and SI cut-off ranges. The latter provides an objective assessment of the myocardial wall substrate and solves intrinsic problems related to selection bias of VT corridors located in one specific layer, but not in others. This strategy also overcomes some limitations of endocardial voltage mapping, which is limited to ≈1 mm deep from the endocardial side.^17^ And third, procedure planning for catheter-based ablation can be substantially simplified in cases aiming at substrate ablation or in those with unmappable VTs during the procedure. In such cases, the target ablation area derived from systematic imaging processing of LGE-CMR images would be substantially smaller (∼50% smaller) than the ablated regions reported with scar homogenization.^23^ Moreover, the systematic imaging processing proposed in this work might also improve procedure planning in patients with complex and refractory VT episodes subject to non-invasive cardiac radiation.^24^ The contribution of novel and more efficient computational models might also help in determining the most relevant imaging-based corridors based on the prevalent VT morphology.^25^

Current commercially available software tools enable clinical electrophysiologists to efficiently process cardiac LGE-CMR images and reconstruct the 3D myocardial substrate.^8,9,26^ More specific tools for scar characterization and imaging integration with electroanatomical mapping systems have also been incorporated into new versions of such software tools after initial research series suggesting incremental clinical value.^9^ Here, we specifically used systematic processing of SI cut-off ranges using the ADAS 3D software. Further processing of the exported data was done using custom MATLAB scripts to provide surface myocardial visualization of CMR-based potential VT corridors. The latter could be easily implemented into new versions of commercial software since our code will be fully available upon request.

The study has some limitations. First, the series was focused on patients with underlying ischaemic cardiomyopathy, which represents a substrate with predominant endocardial involvement of 3D reentrant circuits.^13^ Therefore, new series using the same imaging-based approach are warranted in patients with predominantly epicardial substrates.^27^ Second, further studies are also warranted to address the clinical outcomes of imaging-based guided procedures compared to other substrate-based electrophysiological strategies for VT ablation. Third, electrophysiological- and imaging-derived data were compared after a registration process which is not exempt of small localization errors. Fourth, our analysis did not incorporate information from other imaging modalities which might have provided complementary information to identify a more specific pro-arrhythmic substrate.^28^ Fifth, patients with anticipated poor collaboration during 3D LGE-CMR sequences could have not been included, which may have increased our imaging data quality compared to regular clinical practice. Finally, VT recurrence at 1-year of follow-up should be interpreted in the context of patients with mainly well-tolerated VTs favouring appropriate mapping and ablation.

## Conclusions

Systematic processing of multiple SI cut-off ranges from 3D LGE-CMR images identifies myocardial regions associated with VT isthmus sites and further addresses major gaps for imaging integration into routine procedure planning of patients undergoing VT ablation.

## Supplementary Material

euae244_Supplementary_Data

## Data Availability

All data needed to reproduce the study, custom-written code, and graphical user interfaces are available upon reasonable request to the corresponding author. Individual patient data cannot be shared without permission of the study participants and ethical approval.
