# Supplementary material for: Novel systematic processing of cardiac magnetic resonance imaging identifies target regions associated with infarct-related ventricular tachycardia
Source: Europace. 2024 Sep 19;26(10):euae244. doi: 10.1093/europace/euae244 (PMC11472157; doi:10.1093/europace/euae244)
Supplement: euae244_Supplementary_Data [file euae244_supplementary_data.pdf]

Supplemental Material

Novel systematic processing of cardiac magnetic resonance imaging identifies target regions associated with infarct-related ventricular tachycardia

Anesthesia protocol in pigs

All procedures in pigs were performed under general anesthesia. Anesthesia induction was achieved by intramuscular ketamine injection (15 mg/kg), xylazine (2 mg/kg) and midazolam (0.5 mg/kg). Then, the pigs were intubated and mechanically ventilated with oxygen (fraction of inspired O_2 of 21 %) and anesthesia was maintained with continuous intravenous infusion of ketamine (2 mg/kg/h), xylazine (0.2 mg/kg/h) and midazolam (0.2 mg/kg/h). A continuous infusion of intravenous amiodarone (150 mg/h) was administered during the infarction procedure to decrease the incidence of malignant arrhythmias.

Pig model of myocardial infarction

Pigs underwent percutaneous catheterization of the left anterior descending (LAD) coronary artery to inflate an angioplasty balloon and occlude the artery for 60 minutes. The balloon was inflated either proximal or distal to the first diagonal branch to generate different infarct sizes and variable scar distributions. Unfractionated heparin (300 mg/kg) was also administered at the onset of the instrumentation. In case of ventricular fibrillation during the ischemia protocol, a non-synchronized shock was delivered with a biphasic defibrillator. After 60 minutes of occlusion, the balloon was deflated and a coronary angiogram was recorded to confirm patency of the coronary artery and reperfusion.

Magnetic resonance imaging in pigs

All animals underwent *in vivo* substrate characterization using a Philips Achieva 3T-Tx whole-body scanner equipped with a 32-element and phased-array cardiac coil (Philips Healthcare, Best, The Netherlands), as reported elsewhere.[1, 2] Seven minutes after intravenous contrast injection of 0.2 mmol/kg gadoteric acid (Dotarem, Guerbet, France) 3D late gadolinium enhancement cardiac magnetic resonance (LGE-CMR) images were acquired using an inversion-recovery spoiled turbo field echo (IR-T1TFE) with isotropic resolution of 1.5x1.5x1.5 mm (reconstructed to 0.57x0.57x0.75 mm). Segmented and ECG-gated cine steady-state free precession was also performed to acquire 11-13 contiguous short-axis slices covering the ventricles from the base to the apex to evaluate left ventricular ejection fraction (LVEF) (field of view: 280x280 mm; slice thickness: 6 mm without gap; repetition time: 2.8 ms; echo time: 1.4 ms, flip angle: 45; cardiac phases: 25; voxel size: 1.8x1.8 mm; 3 number of excitations.

Magnetic resonance imaging protocols in patients

Hospital Clínico San Carlos and Hospital La Paz. Scans were performed on a 3T magnetic resonance (MR) system (Philips Achieva, Philips Healthcare, Best, The Netherlands). Whole heart three-dimensional late gadolinium enhancement (LGE) images were acquired in short axis orientation using a phase sensitive inversion-recovery (PSIR) with a spoiled turbo field echo (T1-TFE) sequence (repetition time: 4.23 ms, echo time: 2 ms, flip-angle: 15º). The sequence started seven minutes after intravenous administration of 0.2 mmol/kg of gadoteric acid (Dotarem, Guerbet, France). The entire sequence was ECG and respiratory triggered with a respiratory navigator to compensate for minor volume displacements. The acquired resolution was 1.5x1.5x1.5 mm (reconstructed to 0.70x0.70x0.75 mm). In addition to PSIR acquisition, good scar definition was ensured using a Look-Locker sequence before each 3D acquisition to select the correct inversion time for proper healthy myocardium signal nulling.

Maastricht University Medical Center. Scans were performed on a 1.5T magnetic resonance system (Ingenia, Philips Healthcare, Best, the Netherlands). Three-dimensional LGE images were also acquired in short axis orientation using a PSIR T1-TFE sequence (repetition time: 6.5 ms, echo time: 3 ms, flip-angle: 25º). The sequence started ten minutes after intravenous injection of 0.2 mmol/kg gadobutrol (Gadovist, Bayer Pharmaceuticals, Berlin, Germany). The entire sequence was ECG and respiratory triggered using a respiratory navigator to compensate for 5 mm gating window displacements, and using a compressed-sense factor of 3 and spectral fat pre-saturation. The acquired resolution was 1.6x1.6x1.6 mm (reconstructed to 0.8x0.8x0.8 mm). Good scar definition was ensured using PSIR reconstruction for 3D acquisitions to select the correct inversion time for proper healthy myocardium signal nulling.

Hospital Clínic. Scans were performed on a 3T magnetic resonance system (Magnetom Trio, Siemens Healthcare, Erlangen, Germany) equipped with a cardiac-specific software. Three-dimensional LGE-CMR images were acquired using a PSIR T1-TFE sequence (repetition time: 2.6 ms, echo time: 0.9 ms, flip-angle: 15º). The sequence started seven to ten minutes after intravenous administration of 0.2 mmol/kg gadodiamide-DTPA (Omniscan, Amersham Health). The sequence was also ECG and respiratory triggered with a navigator to compensate for minor volume displacements. Each 3D slice was acquired in a transaxial direction, centered on the area of interest and with a 512x512 pixel matrix. A cartesian trajectory was used to fill the k-space. The voxel size was adjusted to obtain a spatial isotropic resolution of 1.2x1.2x1.2 mm (reconstructed to 1.2x1.2x1.2 mm). Additional scan parameters included a width-band of 801 Hz/pixel. In addition to PSIR acquisition, good scar definition was ensured using a Look-Locker sequence before each 3D acquisition to select the correct inversion time for proper healthy myocardium signal nulling.

Left ventricular ejection fractions were also assessed by multiphase-multislice acquisition with a balanced TFE sequence (1.8x1.8x8.0 mm).

Segmentation of LGE-CMR images

Clinical and experimental LGE-CMR images were processed with the ADAS 3D software (Version 2.11.0, Adas3D Medical S.L., Barcelona). LGE-CMR images were carefully segmented using initial anatomical landmarking and subsequent semiautomatic adjustment of the left ventricular endocardial and epicardial contours. More specifically, segmentations were performed by a first investigator with expertise in cardiac imaging (ARP). Then, a second investigator (JS) (also with expertise in cardiac imaging) further reviewed and corrected, if necessary, the segmentation of the endocardial and epicardial contours. In case of disagreement between the first and second operator, a third investigator (DFR), with large experience in cardiac imaging processing and analysis, reviewed the case to take the final decision. In cases with electrophysiological confirmation of the VT isthmus in the right ventricle, the segmentation was performed manually. Segmentations were not repeated or modified after imaging processing.

Identification of CMR-based potential VT corridors

Segmented images were processed using a systematic approach to obtain imaging-derived potential VT corridors from 15 different and sequential signal intensity (SI) cut-off ranges. Such 15 SI cut-off ranges were selected from the most commonly reported in the literature, starting at 33% (for heterogenous scar) and 53% (for dense scar) of the maximum SI detected with the ADAS3D software. The starting point at 33% was selected after an initial analysis, in which SI cut-off ranges <33%-53% showed minimal contribution (6.06%) to imaging-derived potential VT corridors (Suppl. Figure 2). For each SI cut-off range, the software computed all the potential VT corridors from the endocardium to the epicardium across 9 myocardial layers (i.e., from 10% to 90% of the myocardial wall thickness). The surface projection of VT corridors was performed with the k-nearest neighbors algorithm based on Euclidean distances between 3D vertexes. Overlapping endocardial or epicardial areas of potential VT corridors from different layers were projected (as appropriate) within the same footprint region.

Ventricular tachycardia mapping in pigs

Invasive procedures were performed using endocardial and epicardial access to obtain full anatomical reconstruction of the heart surfaces. The epicardial access was obtained using a conventional subxiphoid approach. The femoral vein and femoral artery were used for percutaneous access to the right and left ventricles, respectively. The femoral artery was also used for continuous monitoring of blood pressure throughout the entire procedure. A screw-in catheter was positioned in the right ventricle for programmed ventricular stimulation. Endocardial and epicardial geometries were generated using the fast anatomical mapping technology of the Carto3 system (Biosense Webster, Diamond Bar, CA) and a 3.5-mm irrigated-tip mapping/ablation catheter (Navistar Thermocool, Biosense Webster). Upon ventricular tachycardia (VT) induction, if the arising VT was hemodynamically stable, the isthmus was localized using activation maps and entrainment maneuvers with a 3.5-mm tip catheter or a 20-pole steerable mapping catheter (Pentaray, Biosense Webster).[3] VT maps, intracardiac electrograms and surface ECG tracings were stored for further offline processing. All electrical and blood pressure recordings were continuously monitored using the LabSystem-Pro electrophysiology recording system (Boston Scientific, Lowell, MA).

Mapping procedure in patients

The invasive electrophysiological study was performed using percutaneous venous and arterial femoral access to reach the right and left ventricles, respectively. Ventricular geometries were generated using the Carto3 (Biosense Webster, Diamond Bar, CA) or the Ensite NavX mapping system (Velocity, Precision or X, Abbott, Abbott Park, Illinois). VT induction was attempted using programmed ventricular stimulation from the right ventricular apex. All stimuli were synchronized with the intrinsic QRS complex. The protocol consisted of two sequential BDCLs of 10 beats each (S1) at 600 or 400 ms. Each BDCL was followed by up to 3 extrastimuli (S2, S3, S4) decremented in CL until reaching refractoriness or a minimum coupling interval of 200 ms. Norephedrine was administered intravenously to support blood pressure if mean arterial pressure was <60 mm Hg. Direct current defibrillation was delivered to restore sinus rhythm in case of ventricular fibrillation or hemodynamic collapse. If the arising VT was hemodynamically tolerated, activation maps and entrainment maneuvers were performed to localize VT isthmus sites. In case of hemodynamic instability during VT mapping, deductive reconstruction of the reentrant circuit associated with the most clinically relevant VT (i.e., documented VT morphology on admission) was performed using pace-mapping.

Delineation of VT regions of interest

VT regions containing the isthmus sites were delineated using at least one of the following mapping data: entrainment, activation maps during VT and pace mapping. Activation maps were color-coded using the QRS onset as the reference for the beginning of the mapping window (Figure 2A). Pacing maps with deductive reconstruction of the reentrant circuit were also color-coded based on the entrance and exit sites (Suppl. Figure 4A).

Registration of electrophysiologically-defined VT regions of interest onto CMR geometries

The registration process started with the identification of specific anatomical landmarks from different spatial planes in both the electroanatomical mapping and CMR geometries. Registration of the VT region of interest onto the right ventricular CMR geometry was performed in cases with confirmation of at least part the VT isthmus in the right ventricle. For the left ventricle, we used the aortic root, the left ventricular apex and the septal and lateral part of the mitral annulus. For the right ventricle, we used the pulmonary artery root, the right ventricular apex and the septal and lateral part of the tricuspid annulus. Then, the registration was done using the mesh registration module of the Amira-Avizo software 2021 (ThermoFisher Scientific, Berlin).

Comparisons between electrophysiologically-defined VT regions of interest and CMR-based potential VT corridors

Electrophysiologically (EP)-defined VT regions of interest (ROI) were visualized on the endocardial surface, or epicardial, if the VT ROI involved the epicardial layers of the left ventricle. The final area of CMR-based potential VT corridors between 10% to 50% of the myocardial wall thickness was color-coded in red on the endocardial CMR geometry (Figure 2C). Likewise, if any, the area of CMR-based potential VT corridors between 60% to 90% of the myocardial wall thickness was color-coded in light-blue on the epicardial CMR geometry. Overlapping endocardial, or epicardial (if applicable), regions between EP-defined VT ROIs and CMR-based potential VT corridors were quantified and color-coded in black (Figure 2C).

Quantification of the ablation area

All mapping and ablation data were exported from the electroanatomical mapping system for further processing using custom Matlab scripts. Ablation lesions were represented on the 3D coordinates of the electroanatomical mesh. The lesions were also visualized on the left ventricular CMR geometry after registration with the electroanatomical mesh. Ablation lesion size was established at 6-mm diameter based on previous data aiming to achieve contiguous lesions.[4] The total ablation area was computed using the lesion size for each lesion. Overlapping ablation lesions were considered on the same ablation area. Ablation lesions <6-mm apart were considered contiguous.[4] Continuity among ablation lesions was represented in dark-grey around the black circles of the ablation lesions (Figure 5B and Suppl. Figure 4). Ablation lesions with <5 seconds of radiofrequency delivery were excluded for quantification of the ablation area.

Supplemental References

[1] Merino-Caviedes S, Gutierrez LK, Alfonso-Almazan JM, Sanz-Estebanez S, Cordero-Grande L, Quintanilla JG, et al. Time-efficient three-dimensional transmural scar assessment provides relevant substrate characterization for ventricular tachycardia features and long-term recurrences in ischemic cardiomyopathy. *Scientific reports* 2021; **11**: 18722.
[2] Lopez-Yunta M, Leon DG, Alfonso-Almazan JM, Marina-Breysse M, Quintanilla JG, Sanchez-Gonzalez J, et al. Implications of bipolar voltage mapping and magnetic resonance imaging resolution in biventricular scar characterization after myocardial infarction. *Europace* 2019; **21**: 163-174.
[3] Anter E, Tschabrunn CM, Buxton AE, Josephson ME. High-Resolution Mapping of Postinfarction Reentrant Ventricular Tachycardia: Electrophysiological Characterization of the Circuit. *Circulation* 2016; **134**: 314-327.
[4] Phlips T, Taghji P, El Haddad M, Wolf M, Knecht S, Vandekerckhove Y, et al. Improving procedural and one-year outcome after contact force-guided pulmonary vein isolation: the role of interlesion distance, ablation index, and contact force variability in the 'CLOSE'-protocol. *Europace* 2018; **20**: f419-f427.

Supplemental Figures

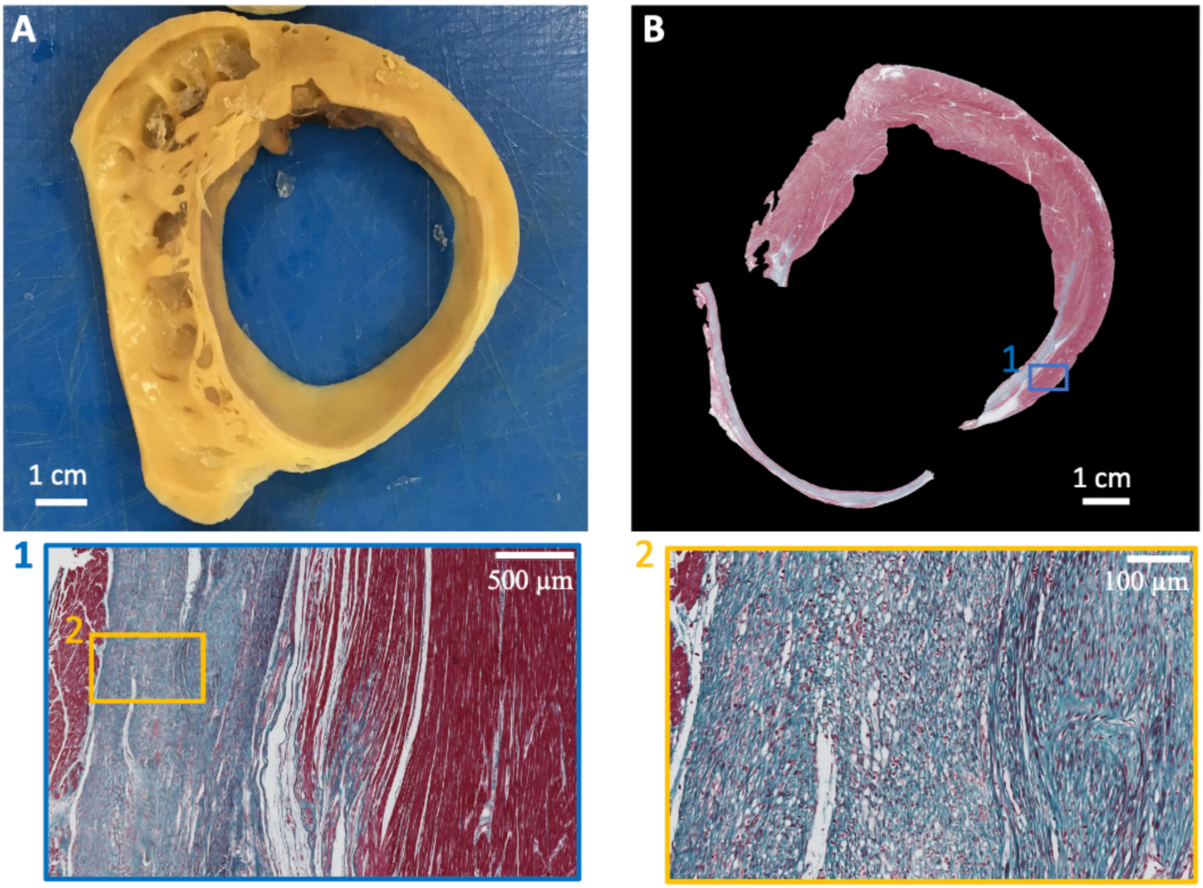

Suppl. Figure 1. Sample left ventricular infarct-related substrate at 12 weeks after ischemia-reperfusion in pigs. A, Macroscopic bright field short axis view of the mid portion of the left ventricle. **B,** Masson's Trichrome staining shows a well-established myocardial infarction in the anterior wall with complete replacement of cardiomyocytes with mature collagen (representative zoom-in view on inset 1; with blue frame). The substrate shows lack of inflammatory cells or granulation tissue (representative zoom-in view on inset 2; with yellow frame).

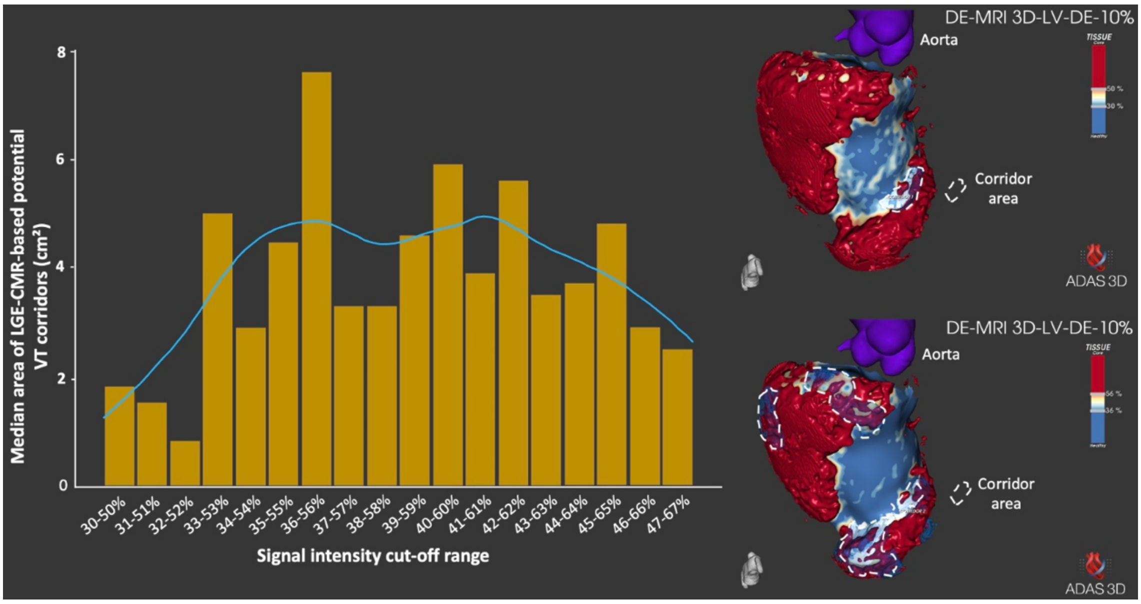

Suppl. Figure 2. Quantification of imaging-derived areas of potential ventricular tachycardia corridors from late gadolinium enhancement cardiac magnetic resonance images in patients. Left, bars graph showing the median area of late gadolinium enhancement cardiac magnetic resonance (LGE-CMR) imaging-derived corridors using different values of signal intensity cut-off ranges in the patient cohort (N=17). The blue line indicates the trend of the median values at each of the signal intensity cut-off ranges. Signal intensity cut-off values <33%-55% were associated with the smallest area (6.06% of the total area) of imaging-derived potential ventricular tachycardia (VT) corridors. A sample case using the signal intensity cut-off range 30-50% and 36-56% is shown on the right. LV: left ventricle.

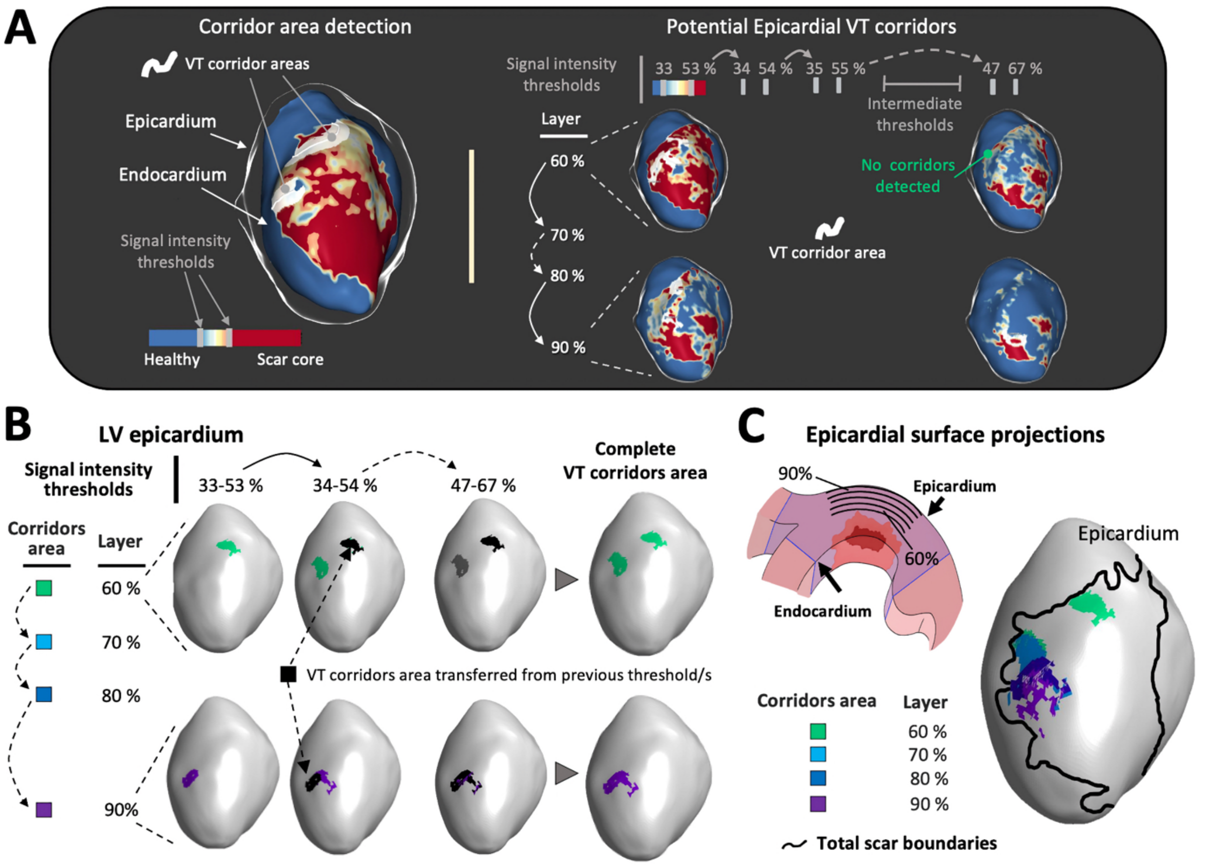

Suppl. Figure 3. Imaging processing for identification of imaging-based potential subepicardial ventricular tachycardia corridors. A, Representative workflow for imaging processing using the ADAS 3D software after semiautomatic segmentation of the left ventricle (LV). All VT corridors (using 15 sequential signal intensity [SI] cut-off ranges) from 60% to 90% of the myocardial wall thickness were exported for further imaging processing using Matlab. **B and C,** All computed subepicardial VT corridors from all SI cut-off ranges were projected on the epicardial surface. Overlapping corridor areas from different subepicardial layers (i.e., 60% to 90% of myocardial wall thickness) or SI cut-off ranges were represented on the same area (C).

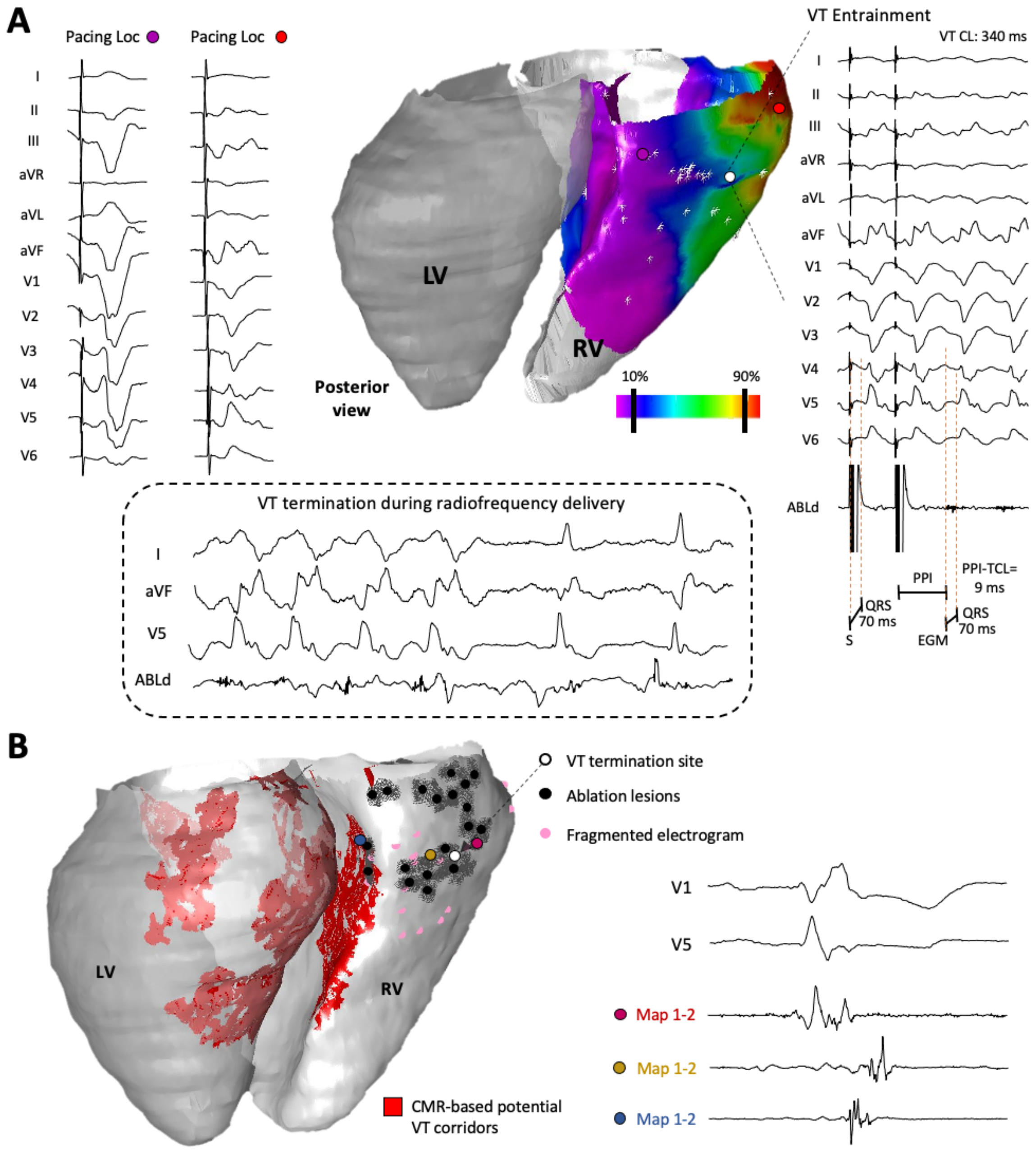

Suppl. Figure 4. Ventricular tachycardia characterization in a patient with ablation targets in the right ventricle. **A,** Sample color-coded pace mapping and entrainment maneuvers to identify the isthmus site in a patient with infarct-related substrate affecting the left and right ventricle. Ventricular tachycardia (VT) termination was documented upon radiofrequency delivery at the distal curvature (exit) of the reentrant circuit (white circle with black border). **B,** Additional ablation lesions were delivered at the proximal curvature (entrance) and common channel (midisthmus), which were localized on the posterior-basal part of the right ventricular septum and the posterior-basal portion of the posterior wall of the right ventricle, respectively. The overlapping region between the cardiac magnetic resonance-based potential VT corridors and the reentrant circuit was in the entrance site of the isthmus (blue circle). This may show some limitations of the imaging processing to detect imaging-based VT corridors in the right ventricular wall. Activation during sinus rhythm showed late potentials also compatible with a VT channel. Sample late potentials are shown with red-, yellow- and blue-filled circles. Pink circles represent locations with fragmented electrograms. Ablation lesions are shown in black circles surrounded by dark-grey.

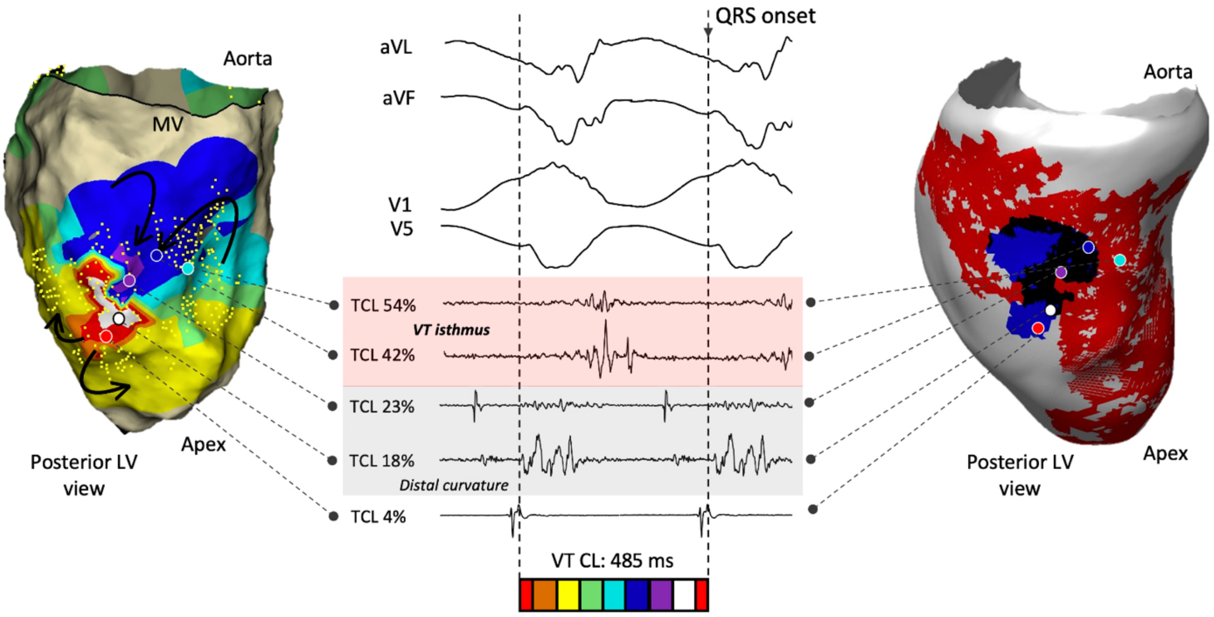

Suppl. Figure 5. Sample electrophysiological characterization of a ventricular tachycardia circuit during activation mapping and spatial correlation with the infarct-related substrate derived from late gadolinium enhancement cardiac magnetic resonance imaging. Left, Activation map of the ventricular tachycardia (VT) morphology shown in Figure 4B (Main manuscript). Color-coded dots indicate different electrogram (EGM)-QRS times and their relative (%) activation time respect to the tachycardia cycle length (TCL) (Middle). Right, Spatial positioning of color-coded EGMs on the endocardial surface of the left ventricle (LV) with the corresponding potential VT corridors (in red) derived from systematic imaging processing of 15 signal intensity cut-off ranges. The electrophysiologically-defined VT region of interest is shown in blue and the overlapping region between the imaging derived corridors and the VT isthmus sites is shown in black.

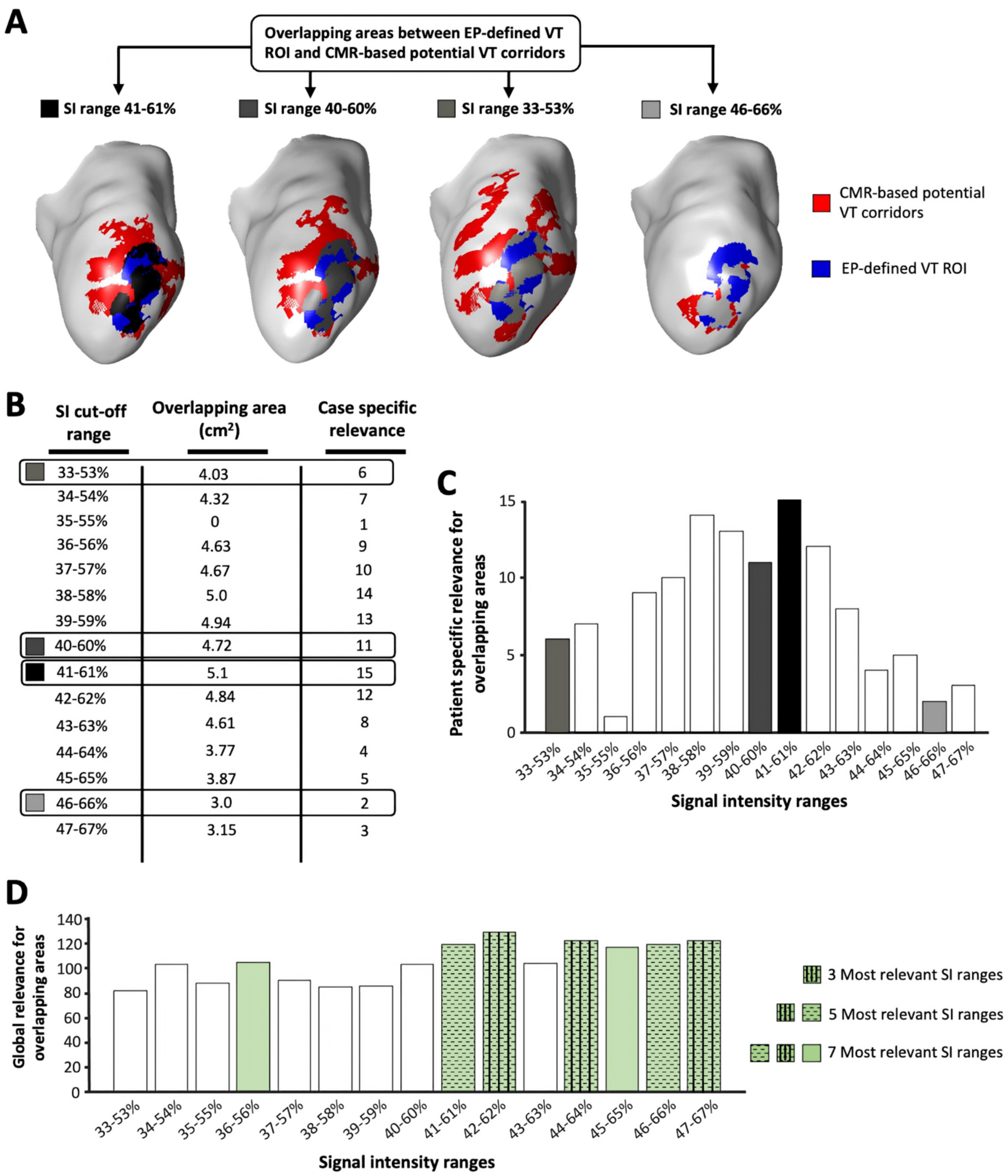

SI cut-off range	Overlapping area (cm²)	Case specific relevance
33-53%	4.03	6
34-54%	4.32	7
35-55%	0	1
36-56%	4.63	9
37-57%	4.67	10
38-58%	5.0	14
39-59%	4.94	13
40-60%	4.72	11
41-61%	5.1	15
42-62%	4.84	12
43-63%	4.61	8
44-64%	3.77	4
45-65%	3.87	5
46-66%	3.0	2
47-67%	3.15	3

Suppl. Figure 6. Determination of the 7, 5 and 3 most relevant signal intensity cut-off ranges among patients. **A,** Sample case showing cardiac magnetic resonance (CMR)-based potential ventricular tachycardia (VT) corridor areas using 4 different signal intensity (SI) cut-off ranges. The electrophysiologically (EP)-defined VT region of interest (ROI) is shown in blue. The overlapping regions between the EP-defined VT ROI and CMR-based potential VT corridors are shown from dark-grey to light-grey colors, depending on the SI cut-off range. **B,** Quantification of the relevance of each of the 15 SI cut-off ranges used for systematic imaging processing for the case shown in A. The relevance score is based on a scale from 1 to 15, in which the highest score is assigned to the SI cut-off range providing the largest overlapping area between the EP-defined VT ROI and the CMR-based potential VT corridors. **C,** Bar chart with the specific score for each SI cut-off range for the case shown in A. Bars with dark-grey to light-grey colors indicate the SI cut-off ranges shown in A. **D,** Overall quantification of the relevance of each of the 15 SI cut-off ranges used for systematic imaging processing among patients. The 7, 5 and 3 most relevant SI cut-off ranges are shown with green bars and specific background grids.

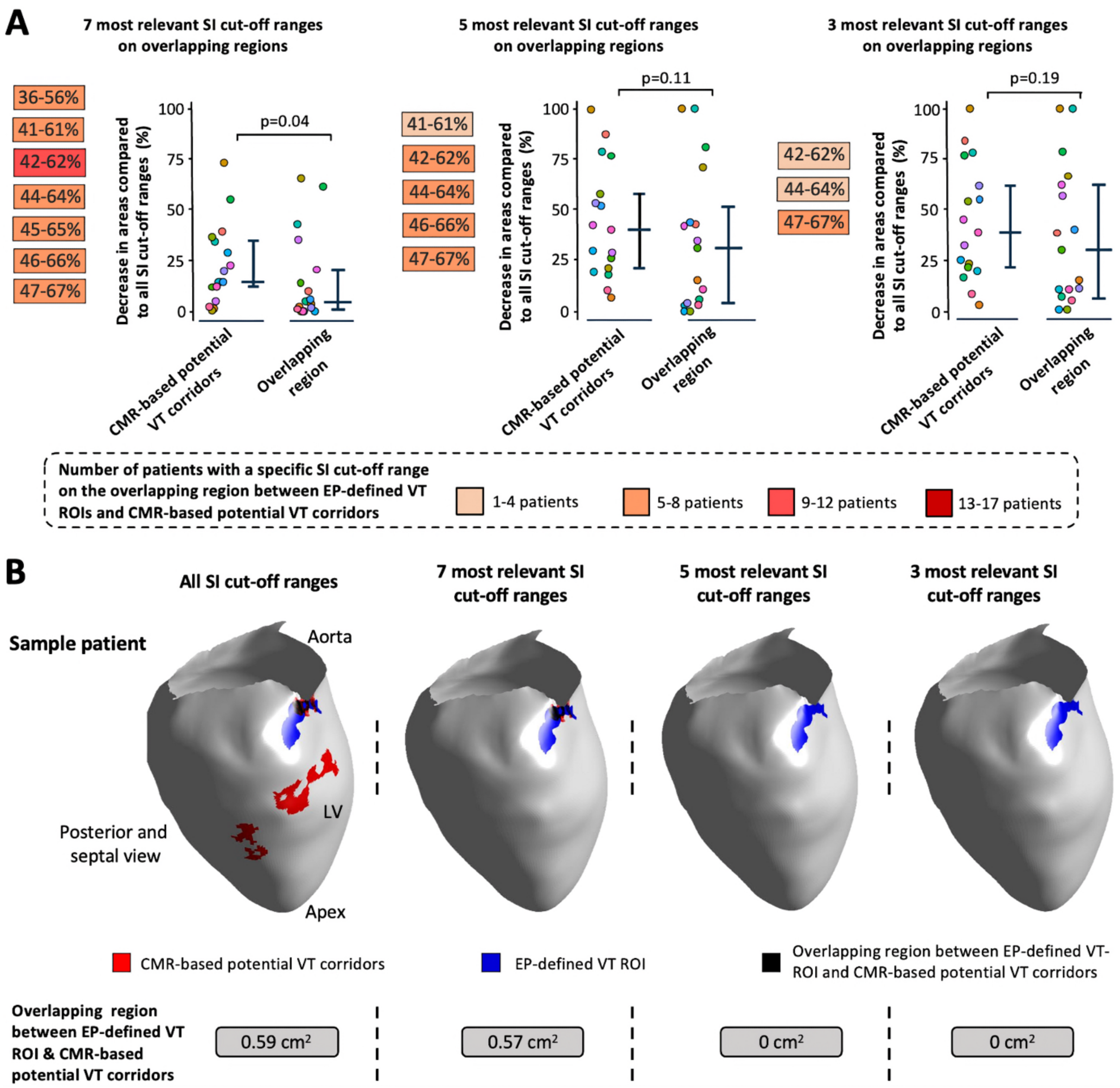

Suppl. Figure 7. Effects of decreasing the number of signal intensity cut-off ranges for systematic imaging processing in patients. A, Representation of the 7, 5 and 3 most relevant signal intensity (SI) cut-off ranges among the 17 patients included in the analysis. Quantification of the decrease in areas of cardiac magnetic resonance (CMR)-based potential ventricular tachycardia (VT) corridors using the 7, 5 and 3 most relevant SI cut-off ranges compared to all (15) SI cut-off ranges. The same quantifications are shown for the overlapping regions between the area of electrophysiological (EP)-defined VT regions of interest (ROIs) and CMR-based potential VT corridors. Each color-coded circle represents the data from a specific patient. **B,** Sample case showing the effects of decreasing the number SI cut-off ranges on CMR-based potential VT corridors (in red), and on the overlapping region (in black) between the EP-defined VT ROI (in blue) and the CMR-based potential VT corridors.

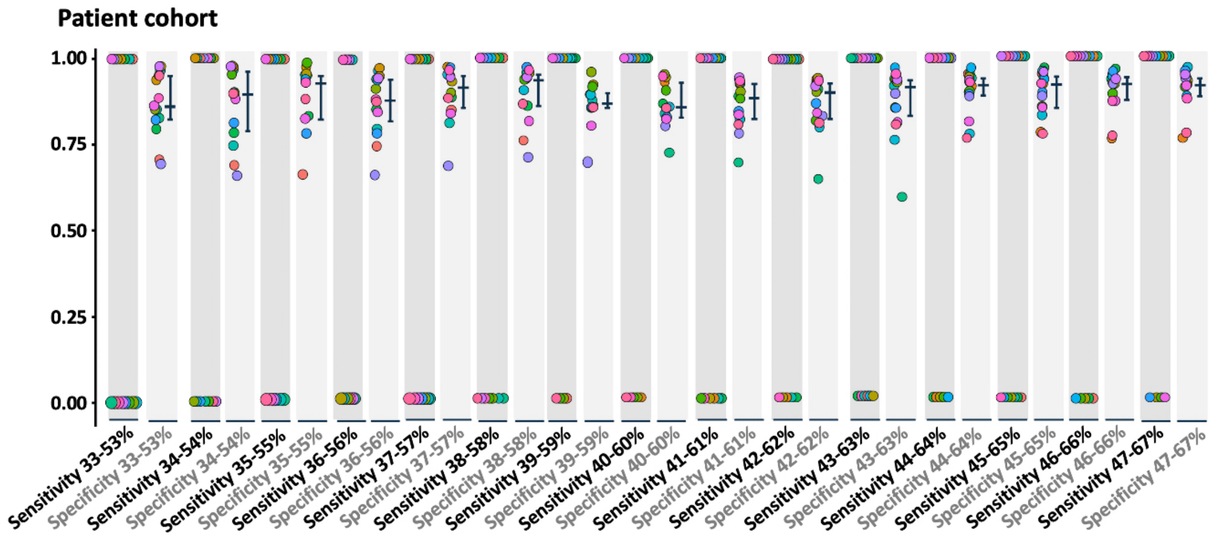

Suppl. Figure 8. Sensitivity and specificity values of single signal intensity cut-off ranges to detect functionally relevant ventricular tachycardia regions containing isthmus sites in patients with infarct-related substrate undergoing mapping and ablation. None of single signal intensity cut-off ranges on 3D late gadolinium enhancement cardiac magnetic resonance images provided 100% sensitivity. This nulls the possibility to detect on imaging-derived corridors from single signal intensity cut-off ranges the electrophysiologically-defined ventricular tachycardia regions of interest (containing isthmus sites) for all patients.

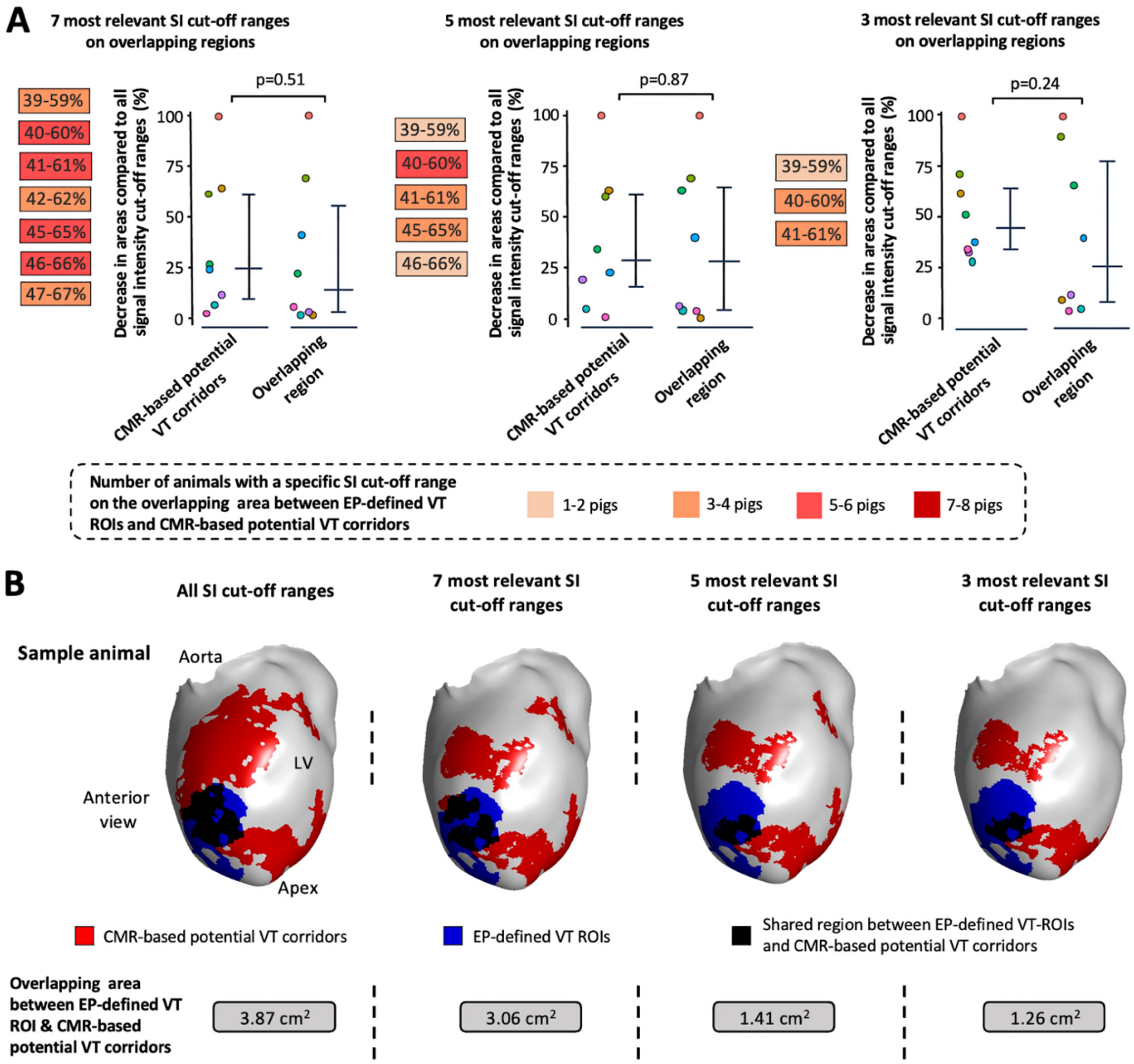

Suppl. Figure 9. Effects of decreasing the number of signal intensity cut-off ranges for systematic imaging processing in pigs. A, Representation of the 7, 5 and 3 most relevant signal intensity (SI) cut-off ranges among the 8 animals included in the analysis. Quantification of the decrease in areas of cardiac magnetic resonance (CMR)-based potential ventricular tachycardia (VT) corridors using the 7, 5 and 3 most relevant SI cut-off ranges compared to all (15) SI cut-off ranges. The same quantifications are shown for the overlapping regions between the area of electrophysiological (EP)-defined VT regions of interest (ROIs) and CMR-based potential VT corridors. Each color-coded circle represents data from a specific pig. **B,** Sample animal case showing the effects of decreasing the number SI cut-off ranges on the CMR-based potential VT corridors area (in red), and on the overlapping region (in black) between the EP-defined VT ROI (in blue) and the CMR-based potential VT corridors.

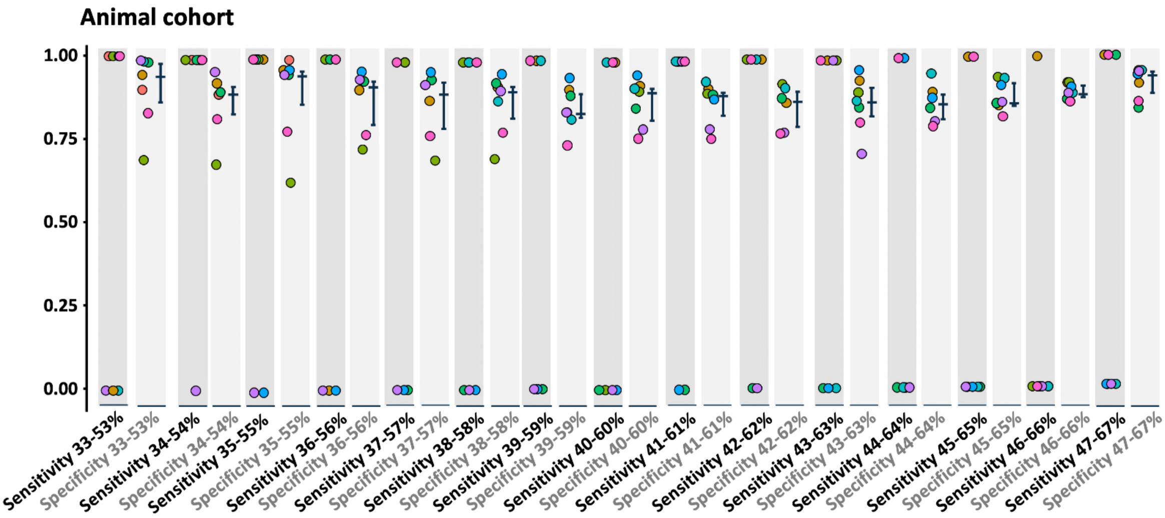

Suppl. Figure 10. Sensitivity and specificity values of single signal intensity cut-off ranges to detect functionally relevant ventricular tachycardia regions containing isthmus sites in pigs with infarct-related substrate undergoing invasive mapping. None of single signal intensity cut-off ranges on 3D late gadolinium enhancement cardiac magnetic resonance images provided 100% sensitivity. This nulls the possibility to detect on imaging-derived corridors from single signal intensity cut-off ranges the electrophysiologically-defined ventricular tachycardia regions of interest (containing isthmus sites) for all animals.

Suppl. Table 1. Baseline animal characteristics and electrophysiological data.

	**Animals (N=8)**
**Baseline characteristics**	
Male, n (%)	8 (100.0)
Age (months)	6 (5, 6)
Weight (kg)	60.2 (54.4, 64.0)
Left ventricular ejection fraction, %	33.9 (30.8, 37.5)
**VT characterization**	
LGE-CMR imaging before VT mapping, days	3 (2.5, 3)
3D LGE-CMR acquisition*, min	8.6 (7.3, 9.2)
Inducible VTs, n	19
VT morphologies, n	2.5 (1.0, 3.5)
VT cycle length, ms	244 (207.2, 285.0)
Mapping strategy for characterization of the VT circuit	
Activation map, n (%)	11 (100)
Entrainment, n (%)	3 (27.2)
Valid mapping points on activation maps, n	648 (337, 755)

Values are expressed as median and interquartile ranges and n (%), as appropriate. LGE-CMR: late gadolinium enhancement cardiac magnetic resonance; *3D LGE-CMR acquisition excludes the time from contrast administration to the beginning of imaging acquisition (7 minutes). VT: ventricular tachycardia.
